# Structural Dynamics of the *Bacillus subtilis* MntR Transcription Factor Is Locked by Mn^2+^ Binding

**DOI:** 10.3390/ijms24020957

**Published:** 2023-01-04

**Authors:** Zoe Jelić Matošević, Katarina Radman, Jolene Loubser, Ivo Crnolatac, Ivo Piantanida, Ignacy Cukrowski, Ivana Leščić Ašler, Branimir Bertoša

**Affiliations:** 1 Department of Chemistry, Faculty of Science, University of Zagreb, Horvatovac 102a, HR-10000 Zagreb, Croatia; 2 Department of Chemistry, Faculty of Natural and Agricultural Sciences, University of Pretoria, Lynnwood Road, Hatfield, Pretoria 0002, South Africa; 3 Division of Organic Chemistry & Biochemistry, Ruđer Bošković Institute, Bijenička cesta 54, HR-10000 Zagreb, Croatia; 4 Division of Physical Chemistry, Ruđer Bošković Institute, Bijenička cesta 54, HR-10000 Zagreb, Croatia

**Keywords:** MntR transcription factor, molecular dynamics simulations, manganese metallosensors, protein structural dynamics

## Abstract

Manganese (II) ions are essential for a variety of bacterial cellular processes. The transcription factor MntR is a metallosensor that regulates Mn^2+^ ion homeostasis in the bacterium *Bacillus subtilis*. Its DNA-binding affinity is increased by Mn^2+^ ion binding, allowing it to act as a transcriptional repressor of manganese import systems. Although experimentally well-researched, the molecular mechanism that regulates this process is still a puzzle. Computational simulations supported by circular dichroism (CD), differential scanning calorimetry (DSC) and native gel electrophoresis (native-PAGE) experiments were employed to study MntR structural and dynamical properties in the presence and absence of Mn^2+^ ions. The results of molecular dynamics (MD) simulations revealed that Mn^2+^ ion binding reduces the structural dynamics of the MntR protein and shifts the dynamic equilibrium towards the conformations adequate for DNA binding. Results of CD and DSC measurements support the computational results showing the change in helical content and stability of the MntR protein upon Mn^2+^ ion binding. Further, MD simulations show that Mn^2+^ binding induces polarization of the protein electrostatic potential, increasing the positive electrostatic potential of the DNA-binding helices in particular. In order to provide a deeper understanding of the changes in protein structure and dynamics due to Mn^2+^ binding, a mutant in which Mn^2+^ binding is mimicked by a cysteine bridge was constructed and also studied computationally and experimentally.

## 1. Introduction

The homeostasis of transition metal ions in living cells is controlled by sensitive mechanisms. Transition metal ions participate in a vast array of biochemical processes as structural or catalytic components of biomolecules. Therefore, it is crucial for all living cells to import sufficient amounts of essential transition metal ions. However, due to their chemical reactivity, these ions can also be toxic for the cell [1]. Thus, the cellular concentrations of transition metal ions are tightly regulated by various mechanisms. In bacteria, the most common mechanism for regulating transition metal ion homeostasis is via metallosensors—transcription factors whose DNA-binding affinity is modulated by the binding of their cognate metal ions [2]. The metal ion binding site affinities of these transcription factors are carefully tuned so that the cellular concentrations of metal ions, which are regulated by these proteins, are kept in a narrow physiological range [3].

MntR belongs to the DtxR/MntR family of metallosensors, which consists of Fe^2+^-sensing transcription factors such as DtxR and IdeR, and of Mn^2+^-sensing transcription factors such as SloR, ScaR and MntR [4,5,6]. Proteins from the DtxR/MntR protein family function as homodimers and recognize imperfect DNA palindromes, each subunit of the dimer binding to one side of the palindrome. When the concentrations of the cognate metal ion (either Fe^2+^ or Mn^2+^) are high, the ion binds to the metal ion binding sites, increasing the protein’s DNA-binding affinity [4,7]. Proteins from the DtxR/MntR family thus bind to DNA as transcription repressors—when metal ion concentrations are high, they repress the transcription of the key proteins of metal ion import systems. They all share a common protein fold—they consist of an N-terminal DNA-binding domain, a central dimerization domain and a C-terminal FeoA domain whose function is still not completely clear [4].

The MntR protein from the bacterium *Bacillus subtilis* is known to negatively regulate the expression of the manganese importers MntH and MntABCD. However, it has also been shown to upregulate the transcription of manganese importers MneP and MneS by directly binding to their regulatory regions [8]. Thus, MntR functions both as a transcriptional repressor and as a transcriptional activator.

Unlike other members of this protein family, MntR from *Bacillus subtilis* lacks the C-terminal FeoA domain. Instead, the C-terminal alpha-helices from both monomers cross over to stabilize the dimeric form of the protein. MntR is a dimer in solution regardless of the presence of Mn^2+^ ions. The dimerization domains of the two monomers are connected by hydrophobic interactions, hydrogen bonds and ion pairs [9].

The metal ion binding affinities of MntR follow the Irving–William series, with the Zn^2+^ ion having the highest binding affinity for MntR, followed by Cd^2+^, Ni^2+^ and Co^2+^, and then Mn^2+^ [10,11]. Of these, only Mn^2+^ and Cd^2+^ ions fully activate the protein for DNA binding [10,12]. This indicates that the specificity of metal sensing by the MntR protein is not determined by binding site affinity, but by its specificity. Crystal structures of MntR in complex with Zn^2+^ [11], Cd^2+^ [11], Co^2+^ [13], Mn^2+^ [11], Fe^2+^ [13] and Ca^2+^ [11] have been published. Most of these ions only bind to one of the two metal binding sites in MntR. There are four different ions for which a structure with both sites occupied has been published: Fe ^2+^, Ca^2+^, Mn^2+^ and Cd^2+^. However, the second site in the Fe^2+^–MntR complex is partly solvent-exposed, and likely not physiologically relevant [13]. As for the Ca^2+^–MntR complex, the binding affinity of Ca^2+^ for MntR is too low for it to be physiologically relevant [11]. Thus, only the ions which activate MntR for DNA binding—Cd^2+^ and Mn^2+^—have been shown to bind two ions per monomer in a physiologically relevant concentration range and position. Mn^2+^ binds to MntR in a binuclear binding site, with the two ions 4.4 Å apart [11]. It has been proposed that the first binding site acts as a selectivity filter—the binding coordination in the first binding site determines whether the second site will be occupied [11]. The second binding site is coordinated by residues from the N-terminal portion of the protein (Asp7) and the dimerization domain (Glu99, Glu102, His103). The second binding site is thus likely crucial for properly orienting the DNA-binding domains in regards to the dimerization domain, to enable DNA binding [4].

What is striking about the activation of MntR by Mn^2+^ is the lack of a large conformational difference between the crystal structures of the active Mn^2+^–MntR complex and the significantly less active Zn^2+^–MntR complex [11]. The RMSD between the two crystal structures is 1.65 Å [11]. A comparison of metal-bound crystal structures with crystal structures of the protein in its apo form indicated a larger diversity of protein conformations accompanied by a larger distance between the DNA-binding domains [14].

Conformational changes induced by metal ion binding have also been studied using ANS fluorescence, circular dichroism and deuterium exchange mass spectrometry. Thermal denaturation studies using circular dichroism showed that the protein undergoes significant thermal stabilization upon binding of metal ions—Mn^2+^, Co^2+^, Ni^2+^ and Cd^2+^. For Mg^2+^ and Ca^2+^ ions this effect could not be observed [15]. Deuterium exchange experiments followed by protease digestion on apo-MntR and the Co^2+^–, Cd^2+^– and Mn^2+^–MntR complexes provided a closer look into the conformational changes caused by metal ion binding. Surprisingly, the DNA-binding domain, whose position varied the most in the apo crystal structures, showed low rates of proton exchange, indicating internal rigidity. The linker helix, however, which connects the DNA-binding and dimerization domains, was rigidified by metal ion binding. Other interesting regions were residues 86–92 and 113–115, as they were more rigid in the active forms of the protein (in complex with Mn^2+^ and Cd^2+^) than in the inactive forms (apo-protein and Co^2+^ complex) [16].

In the presented study, combined computational and experimental research was conducted in order to study changes in the structure and dynamics of the of *Bacillus subtilis* MntR protein caused by Mn^2+^ ion binding. Molecular dynamics simulations accompanied by DFT calculations provided insight into structural and dynamic properties of different forms of the MntR protein which were experimentally verified with circular dichroism and differential scanning calorimetry measurements. The presented results contribute to a deeper understanding of the allosteric mechanism of *B. subtilis* MntR.

## 2. Results

### 2.1. Conformational Space of Bacillus subtilis MntR Is Reduced by Mn^2+^ Binding

**Figure 1:**
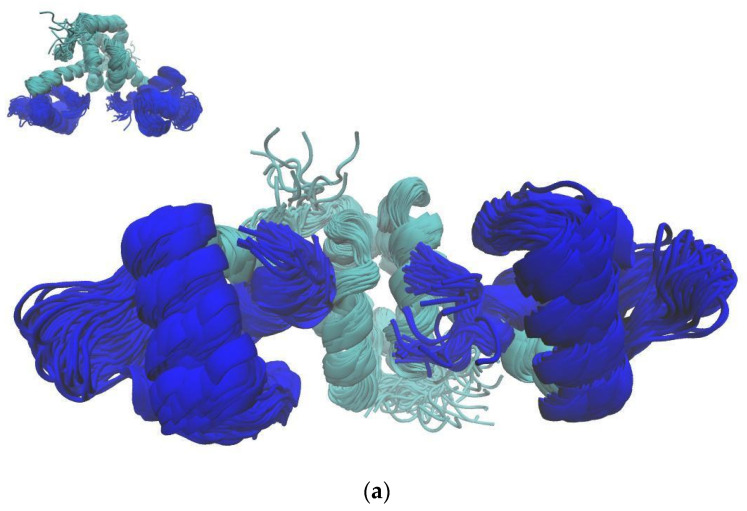

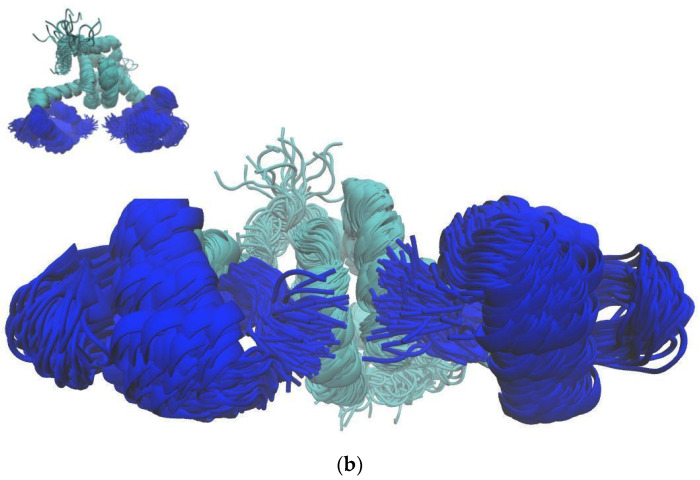
The superposition of snapshots taken every 5 ns during MD simulations of the MntR protein in (**a**) its Mn^2+^-bound form and (**b**) its apo state. Two different orientations of the protein are shown. DNA-binding domains (DBD) are colored blue.

Results of molecular dynamics (MD) simulations show that the conformational space of MntR in its metal-free state is much broader than for the Mn^2+^-bound protein. This is most visible for the DNA-binding domains of the protein, which adopt a wide range of positions in the metal-free state, while they are less mobile in the Mn^2+^-bound state of the protein (Figure 1). Also, visualization shows that most of the conformations that the Mn^2+^-bound protein adopts are also available to the apo-protein, but less populated. 

The differences in the conformational spaces of metal-bound and metal-free forms of MntR were further explored with cluster analysis. A larger number of clusters in the case of the metal-free form compared to the metal-bound form of the protein using the same cut-off confirms a larger conformational space of the metal-free form of the protein (Table 1). Visualization showed that the conformations of the Mn^2+^-bound protein are also available to the apo-protein, but are less populated.

This suggests that the allosteric mechanisms by which manganese ion binding modulates MntR’s DNA-binding affinity is likely not based on a switch between an active and an inactive conformation, but rather on a reduction in the conformational space that favors the DNA-binding conformers. The larger mobility of the DNA-binding domains in the apo-simulations can also be inferred from the residue-wise fluctuation plot (Figure S1 in SI). This is in agreement with experimental data available in the literature, which showed that Mn^2+^ ion binding induces thermal stabilization of the MntR protein using circular dichroism spectroscopy [16].

**Figure 2:**
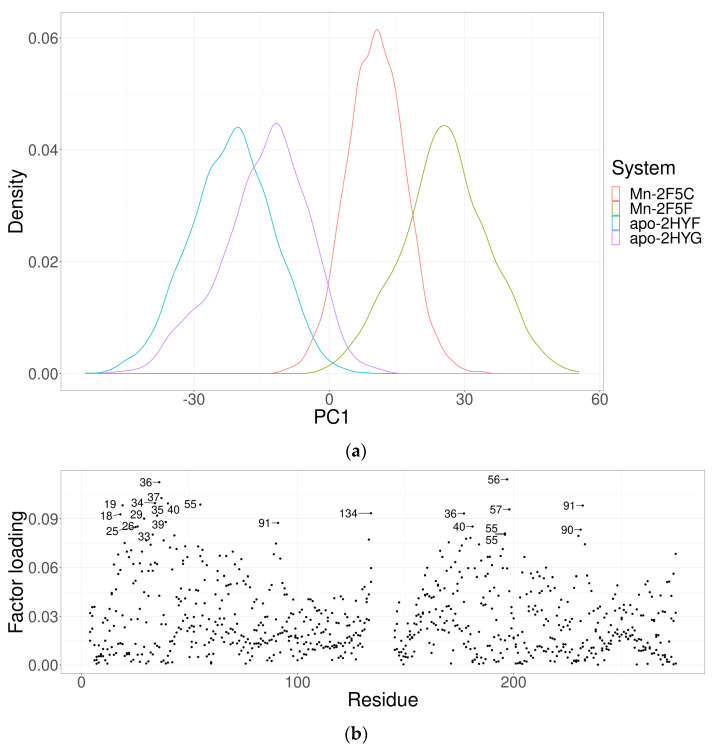

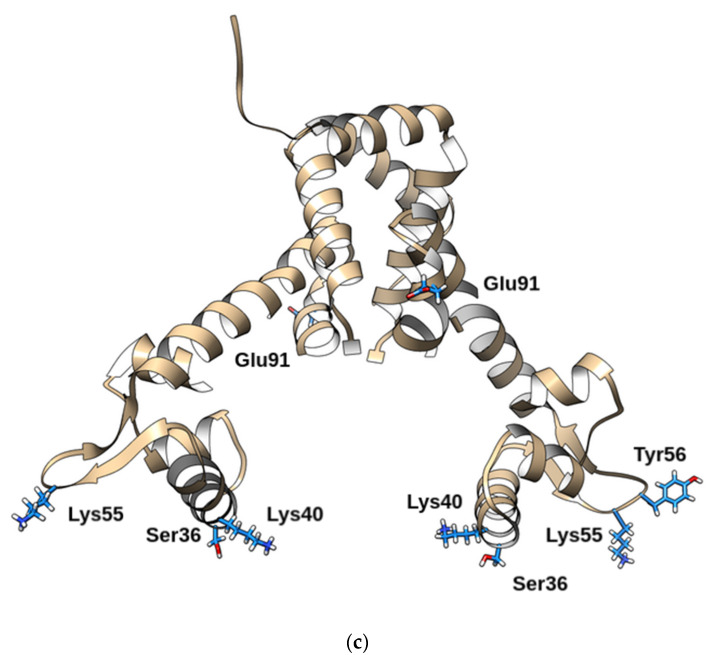
Principal component analysis. (**a**) The projection of four trajectories of the MntR protein in its apo- (red and green) and Mn^2+^-bound forms (blue and purple) onto principal component 1 obtained with principal component analysis of the Cartesian coordinates of the Cα backbone protein atoms. (**b**) Factor loading for principal component 1—the labeled points are counted for each chain of the dimer individually. (**c**) Residues represented in the factor loading for both chains are shown in sticks.

Principal component analysis was performed on the Cartesian coordinates of the protein backbone Cα atoms for equilibrated systems (the last 300 ns of each MD simulation). The first principal component describes 86% of the variance in all simulations, while the second describes 8% and the third principal component describes 3% of the total variance. The projection of the apo- and Mn^2+^-trajectories on the first principal component (PC1) shows a clear separation of PC1 into apo- and Mn^2+^-systems (Figure 2a). The variance in PC1 is thus a reflection of the differences between the apo- and holo-simulations. The factor loadings of PC1 show that the main contributors to these differences are residues within the DNA-binding helices and, surprisingly, Glu91 from the otherwise rigid dimerization domain (Figure 2b,c). Residues Ser36 and Lys40, which appear in both chains, belong to the DNA-binding helix and might be important for specific protein–DNA interactions (Figure 2c). Surprisingly, residues that participate in Mn^2+^-binding are not among the main contributors to PC1. This implies that the difference between the Mn^2+^ and apo systems is not mainly in the Mn^2+^-binding sites, but is allosterically transferred to other parts of the protein. The projection of the trajectories on principal component 2 shows no separation between the apo and Mn^2+^ systems (Figure S2 in SI).

### 2.2. Manganese Ion Binding Locks DBH in Favorable Orientation for DNA Binding

**Figure 3:**
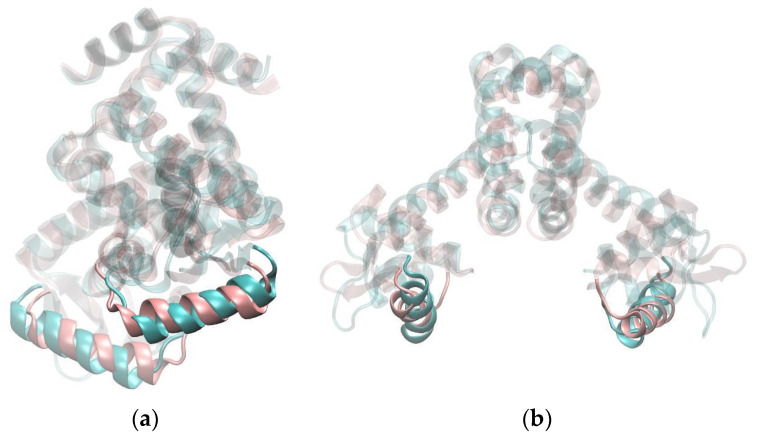
Superposition of the centroids of the most-populated clusters of MD trajectories of apo– and Mn^2+^–MntR systems. The apo system is shown in blue, while the Mn^2+^ system is shown in pink. Two different orientations of the protein (**a**,**b**) are shown.

The most noticeable difference between the apo and the Mn^2+^ simulations is the position of the DNA-binding helices. The results presented in the previous chapter support the hypothesis that the allosteric mechanism of MntR is governed primarily by a reduction in the conformational space due to Mn^2+^ binding, rather than by a conformational switch. Visualization of the trajectories shows that the apo system rarely adopts an orientation of the DNA-binding helices similar to the one in the Mn^2+^ system. The cluster analysis of trajectories confirms that the difference between the apo and the Mn^2+^ systems is most pronounced in the positions and orientations of the DNA-binding helices (Figure 3). In the Mn^2+^ simulations they are closer to each other and have a different orientation than in the case of the apo simulations.

**Figure 4:**
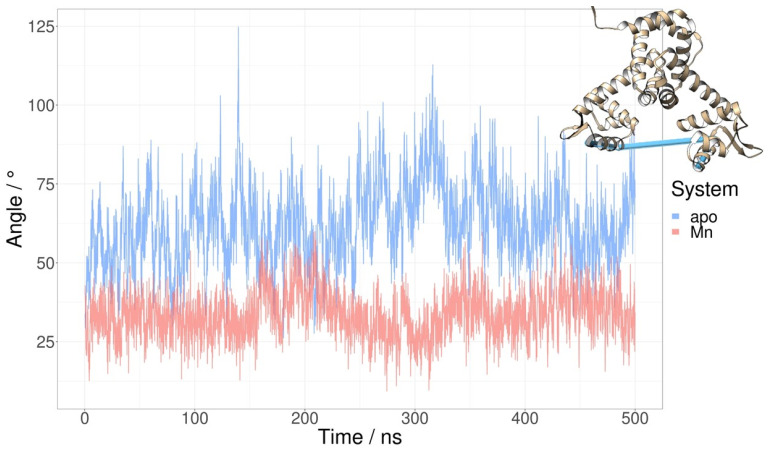
The dihedral angle between the DNA-binding helices for the MntR protein during the MD simulation of apo- (blue) and Mn^2+^-bound form (pink) of the protein. The dihedral angle is defined using the backbone C atoms of residues 47, 36, 189 and 178.

In order to quantify the difference in relative orientation of the DNA-binding helices the dihedral angle between the backbone C atoms of residues 47, 36, 189 and 178 was monitored through MD simulations (Figure 4). The average value of the dihedral angle between the DNA-binding helices is significantly larger for the apo simulation (64.6 ± 12.5° than for the Mn^2+^ simulation (36.5 ± 7.3°). The significantly larger standard deviation of the angle supports the hypothesis that the binding of Mn^2+^ ions reduces the conformational space of the protein and locks the orientation of the DNA-binding helices. 

The observed differences in the dihedral angle between the DNA-binding helices were not mirrored by other, previously used measures, such as the distance between the DNA-binding domains (Figure S5 in SI). The previously described hinge-like motion of the linker helix [14], which is centered at residue 75, was quantified by measuring the angle between the C atoms of residues 64, 75 and 86 (see Section 2.8). This measure also was not appropriate for showcasing the differences between various simulated systems. The same holds for the distance between the Cα atoms of residue Lys41 in the two monomers (Figure S8 in SI).

The interactions of the first coordination shell of the Mn^2+^ ions were parametrised using bonded force field parameters obtained using DFT calculations (see Section 4.1 of the Methods). The first Mn^2+^ ion is coordinated by residues Glu11, His77, Glu99 and Glu102, while the second Mn^2+^ ion is coordinated by residues Asp8, Glu99, Glu102, His103 and a water molecule (Figure S6 in SI). The interaction of the second Mn^2+^ ion with the backbone carbonyl atom of Glu99 was not modelled with bonded force field parameters, but the interaction was nonetheless stable during the simulations (Figure S6).

### 2.3. An attempt to Replicate the Binding of a Second Mn^2+^ Ion with a Cysteine Bridge

**Figure 5:**
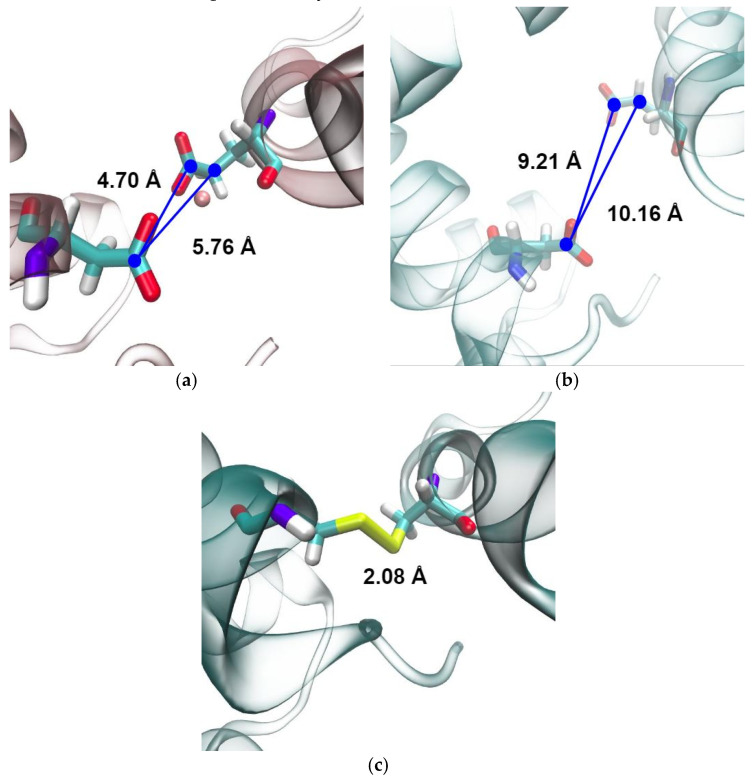
The second binding site of *B. subtilis* MntR. (**a**) The Mn^2+^ ion in the second binding site is coordinated by residues Asp8 and Glu99. (**b**) The distance between Asp8 and Glu99 at the end of the simulation of the apo system. (**c**) The cysteine bridge between residues Cys8 and Cys99 was constructed to imitate the binding of the Mn^2+^ ion in the second binding site.

Analyses of MD simulations indicate that Mn^2+^ binding in the second binding site acts as a “glue” that keeps the dimerization and DNA-binding domains in closer proximity, as the binding site residues from different domains are much further apart in apo simulations (Figure S3 in SI). This is in accordance with the literature data [10,11,13,14] which indicates that Mn^2+^ binding to the second binding site is crucial for MntR activation for DNA binding. In order to test this hypothesis, an attempt was made to produce a constitutively active mutant of the MntR protein in which the role of Mn^2+^ binding in the second binding site (but not in the first binding site) was imitated by introducing a cysteine bridge between the two parts of the binding site. Therefore, a double point mutant (D8C and E99C) in which a cysteine bridge was introduced between residues 8 and 99 (Figure 5), which participate in forming the second Mn^2+^ binding site, was constructed and studied in silico and experimentally. 

**Figure 6:**
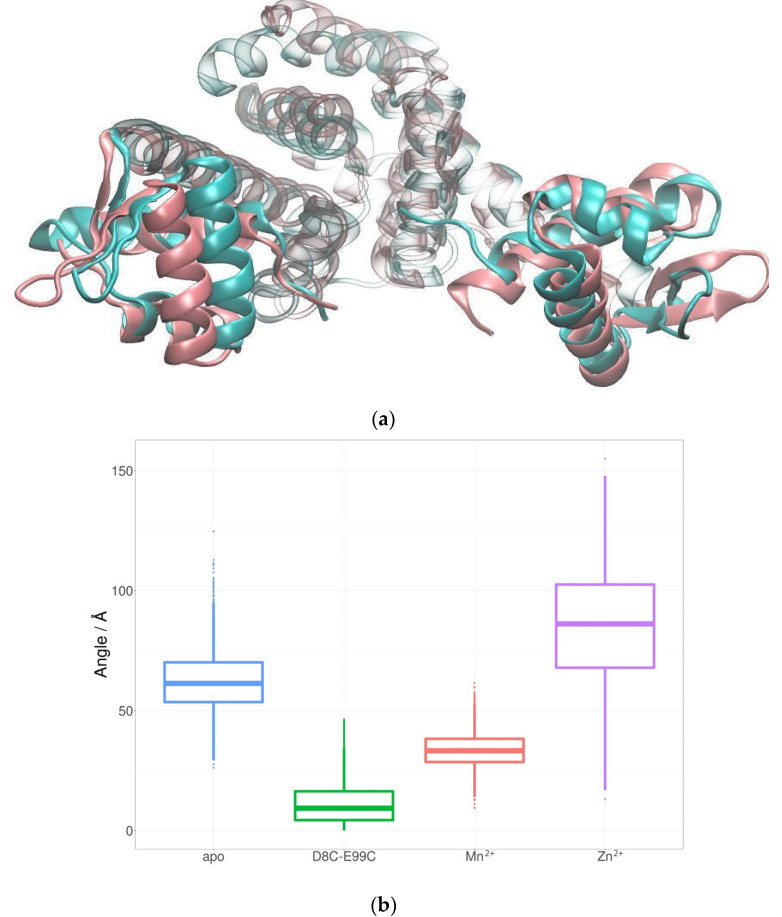
Positions of the DNA-binding helices: (**a**) at the end of the Mn^2+^-bound (pink) and mutant (blue) simulations and (**b**) a boxplot of the dihedral angle between them during the apo (blue), Mn^2+^ (pink), mutant (green) and Zn^2+^ (purple) simulations.

The results of analyses of MD simulations of the D8C-E99C mutant were compared with the results of simulations of the Mn^2+^-bound protein in order to estimate how successfully the mutant mimics Mn^2+^ binding. The dihedral angle between the DNA-binding helices was identified as a promising quantitative measurement of MntR activation (see previous chapter). During MD simulations of the D8C-E99C mutant, the dihedral angle between the DNA-binding helices is much more similar to the values obtained for the simulations of the Mn^2+^-bound system than for the simulations of the apo system (Figure 6). The D8C-E99C mutant was computationally identified as a promising attempt to mimic the Mn^2+^ binding in the second binding site.

### 2.4. Comparison of the Electrostatic Potential and Electrophoretic Mobility of the Wild-Type and D8C-E99C Mutant MntR Protein

**Figure 7:**
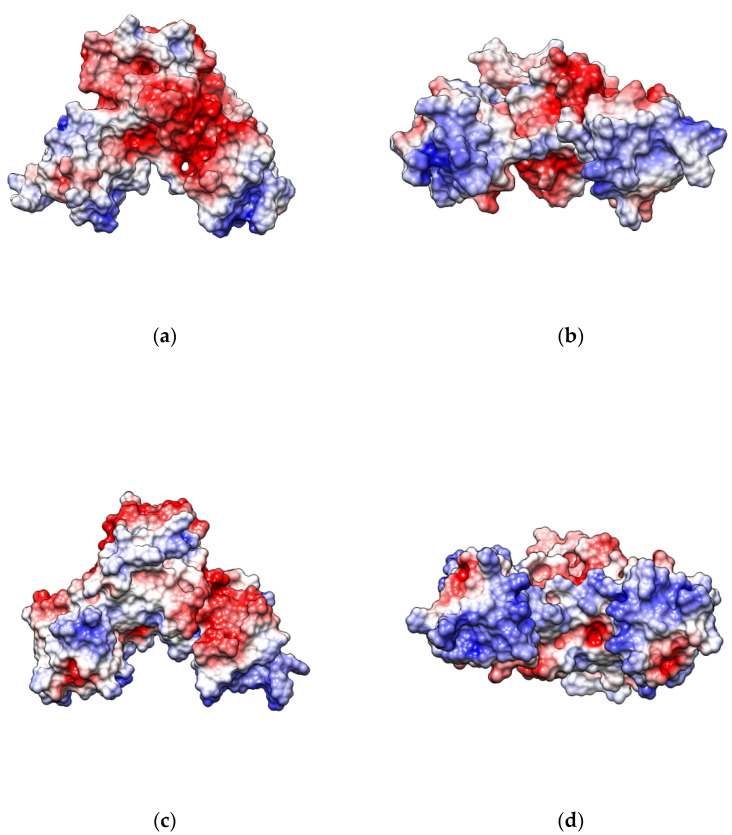

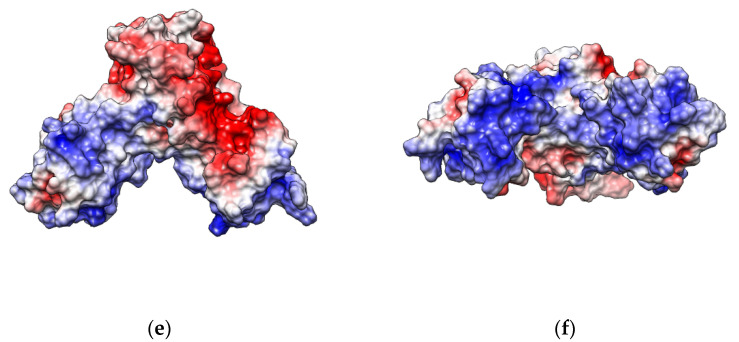
The electrostatic potential on the surface of the MntR protein calculated on the structure which represents the centroid of the most-populated cluster for the simulations of the apo- (**a**,**b**), D8C-E99C mutant (**c**,**d**) and Mn^2+^-bound systems (**e**,**f**). The systems are shown from the front (**a**,**c**,**e**) and bottom (**b**,**d**,**f**). The latter perspective highlights the DNA-binding domain.

The binding of Mn^2+^ ions induces a change in the electrostatic potential of the *B. subtilis* MntR protein. The negative electrostatic potential around the Mn^2+^-binding site, which likely originates from the negatively charged binding site residues, is neutralized by Mn^2+^ binding (Figure 7a,e). At the same time, parts of the dimerization and DNA-binding domains become more positive (Figure 7b,f). In particular, the positive electrostatic potential of the DNA-binding helices is increased in the Mn^2+^-bound systems, likely facilitating binding to the negatively charged DNA molecule (Figure 7b,f).

The electrostatic potential of the mutant is more similar to the apo systems, likely owing to the lack of positively charged Mn^2+^ ions in the binding site. The polarization of the potential on the DNA-binding helices is thus not as pronounced in the mutant as it is in the MntR–Mn^2+^ complex (Figure 7c,d).

Even though the calculated surface electrostatic potential of the D8C-E99C mutant is similar to the one of the wild-type protein in its apo form, a native polyacrylamide gel electrophoresis (native-PAGE) assay demonstrates that the D8C-E99C mutant has a slightly lower electrophoretic mobility than the wild-type protein (Figure S4 in SI). Considering the fact that in native-PAGE proteins are separated according to their size, net charge and conformation, this difference in mobility is likely due to the somewhat more positive overall charge of the mutant which is a consequence of mutating Asp8 and Glu99 to cysteines, combined with an altered secondary structure composition (see chapter below). From this assay it could not be concluded whether either the mutant or the wild-type protein exhibited binding to the recognition sequence in the mntH promotor (5′-AAAATAATTTGCCTTAAGGAAACTCTTT-3′), both in the presence and absence of the Mn^2+^ ion, so in that regard the mutant and the wild-type protein showed similar behavior.

### 2.5. Circular Dichroism of Wild-Type MntR, the D8C-E99C Mutant and Their Complexes with Mn^2+^

**Figure 8:**
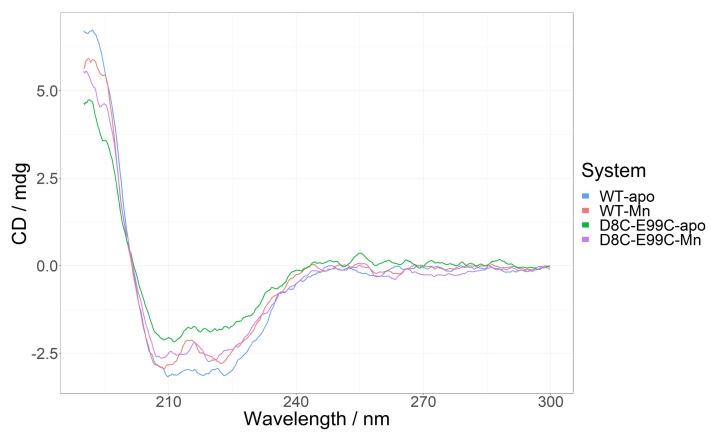
CD spectra in SEC buffer of 40 µM *Bacillus subtilis* apo-MntR in SEC buffer (blue trace) and upon addition of 80 µM of Mn(Ac)_2_ (red trace); and *Bacillus subtilis* apo-MntR D8C-E99C mutant (green trace) and the same sample after addition of 80 µM of Mn(Ac)_2_ (purple trace).

The conformational analysis obtained from CD measurements both for mutant and wild-type protein shows a predominantly helical structure, which upon Mn^2+^ binding becomes even more pronounced. The CD spectra are presented in Figure 8 and the calculated secondary structure motifs are listed in Table 2 below.

### 2.6. Thermally Induced Unfolding of Wild-Type MntR, the D8C-E99C Mutant and Their Complexes with Mn^2+^

**Figure 9:**
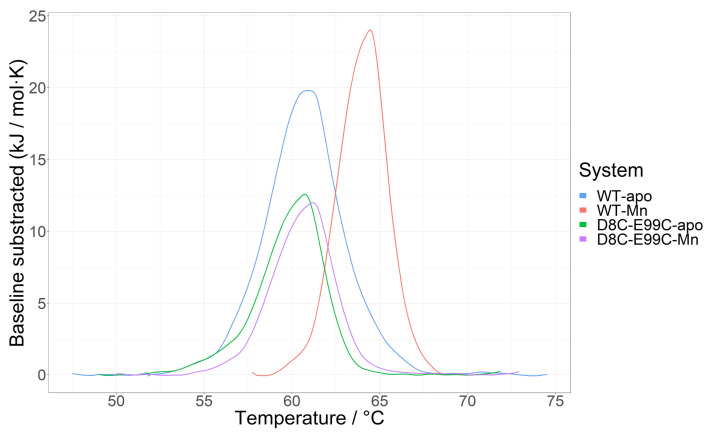
DSC thermograms of apo-MntR (blue trace) and its complex with Mn^2+^ (red trace), as well as the D8C-E99C mutant (green trace) and its complex with Mn^2+^ (purple trace), represented as baseline subtracted molar heat capacity traces. Samples are prepared in SEC buffer; the buffer-buffer scan was subtracted.

DSC thermograms reveal stabilization of the structure upon Mn^2+^ binding, with the ∆T_m_ value 3.3 °C for wild-type protein and 0.42 °C for the mutated one. However, the unfolding of the wild-type MntR-Mn^2+^ complex is a more cooperative process than the unfolding of the apo-MntR wild-type protein. The unfolding peak at ½ of the maximum value of the metal complex shows an almost 20% decrease compared to the apo-protein. The net enthalpy of the unfolding process is therefore somewhat lower with the complex than with apo-MntR (84 kJ/mol vs. 105 kJ/mol, respectively). The introduced D8C-E99C mutation renders a structure that does not change dramatically upon the addition of Mn^2+^ ions. Apart from the slight change in the unfolding temperature, enthalpy change and cooperativity of the unfolding process are all comparable, with negligible differences between apo- and Mn^2+^-complexed MntR D8C-E99C mutant. The thermograms are depicted in Figure 9, while the unfolding parameters are listed in Table 3.

### 2.7. Confirmation of Disulfide Bridge Formation in D8C-E99C Mutant

Ellman’s reagent reaction confirmed the formation of a disulfide bridge between the introduced Cys residues. The measured concentration of free –SH groups was negligible for untreated samples of the wild-type and D8C-E99C mutant *B. subtilis* MntR protein, which by itself indicates that the introduced cysteine amino acids are bound to form a cysteine bridge. Moreover, when protein samples were treated with thiol-free reducing agent, the concentration of free –SH groups was again negligible for the wild-type MntR, while for D8C-E99C mutant it was approximately two times the concentration of the protein (12, 35 and 139 µM for 4.5, 16 and 55 µM protein, respectively).

### 2.8. Simulations of the Singly-Metallated MntR–Zn^2+^ Complex

**Figure 10:**
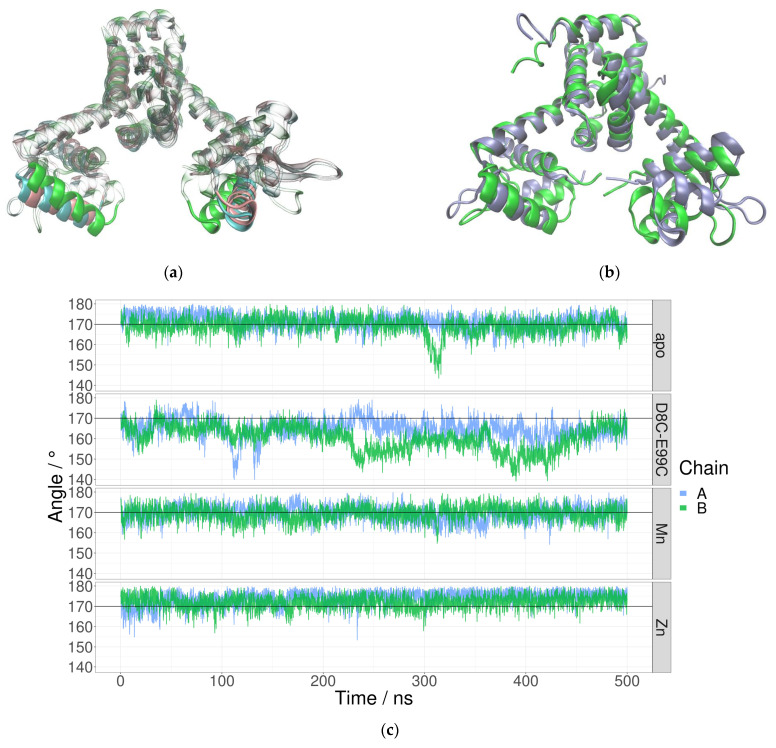
Conformational changes of the Zn^2+^–MntR complex during simulation. (**a**) Superposition of the centroids of the most populated clusters of MD trajectories of apo- (blue), and Mn^2+^–(pink) and Zn^2+^–MntR (green) systems. (**b**) A comparison of the first (0 ns) and final (after 500 ns) structures of the Zn^2+^–MntR complex obtained using MD simulation. (**c**) The angle between the backbone C atoms of residues 64, 75 and 86 was used to quantify the hinge-like motion of the linker helix.

The conformations of the MntR protein in its Zn^2+^-bound state during the MD simulation deviate significantly from the crystal structure of the Zn^2+^–MntR complex (Figure 10). The DNA-binding helices twist away from each other and their positions fluctuate during the simulation even more than in the case of simulation of the apo system (Figure 6). However, this is not accompanied by a larger deviation of the angle at which the linker helix is bent, which is on average similar for all systems: apo (170.3 ± 4.1°), Mn^2+^ (169.5 ± 3.7°) and Zn^2+^ simulations (173.1 ± 3.5°). Since non-bonded parametrization was used, changes in Zn^2+^ coordination were noticed during the simulation, from an initial tetrahedral coordination to an octahedral one (Figure S7 in SI). In the crystal structure, Zn^2+^ is coordinated by residues Glu11, His77, Glu99 and Glu102. During the simulation, an octahedral coordination is achieved—either all three glutamates, Glu11, Glu99 and Glu102, became bidentate ligands, or one of the carboxyl atoms of Glu99 is replaced with a water molecule.

## 3. Discussion

Despite numerous studies and available literature data, the molecular mechanism through which Mn^2+^ binding triggers an increase in the DNA-binding affinity of *B. subtilis* MntR remains a puzzle. The results presented in this paper show the existence of a dynamic equilibrium between the MntR conformations that can bind to DNA and those that cannot. The binding of Mn^2+^ ions shifts this equilibrium towards conformations with higher DNA-binding affinity. Both computational and experimental results support the conclusion about the existence of a dynamic equilibrium between conformations that is shifted by Mn^2+^ binding. 

There is a large number of crystal structures of the MntR protein in complex with various metal ions available. Many ions, such as Zn^2+^ and Co^2+^, only bind to one of the two metal-binding sites of MntR. Although previously published data indicate that this binding mode is unable to fully activate MntR for DNA binding [11], the RMSD between one of the fully active Mn^2+^–MntR complex conformations (PDB ID: 2F5F [11]) and the only partially active Zn^2+^–MntR conformation [11] is only 0.84 Å For comparison, the RMSD between two available structures of the Mn^2+^–MntR complex is 0.89 Å (Table S2 in SI). The four available biological assemblies of the apo form crystal structures show larger conformational diversity than the metal-bound crystal structures, which is in accordance with our results. However, these crystal structures still do not clarify the allosteric mechanism behind the MntR protein, as the RMSD between the two apo-conformations (1.77 Å) is in fact larger than the RMSD between one of the Mn^2+^ structures and the apo structure (1.72 Å) (Table S2 in SI). Crystal structures are static and the significance of the mentioned differences and similarities in conformations can only be fully explored by studying the dynamical behaviour of the apo- and Mn^2+^-bound structures. Therefore, our results enabled a deeper interpretation of the difference in conformational diversity between the apo and Mn^2+^ systems indicated by crystal structures. The results of molecular dynamics (MD) simulations showed that the apo structure has access to a large number of available conformations, while Mn^2+^ binding reduces the conformational space of the protein to a smaller subset of conformations which are adequate for DNA binding (Figure 1). A similar conclusion has previously been reached for AntR, a homologue of MntR from the bacterium *B. anthracis* [17]. Using electron paramagnetic resonance (EPR), Sen et al. have observed a reduction in backbone dynamics of AntR upon Mn^2+^ binding. Since the results obtained for *B. anthracis* AntR were mostly based on experimental research (EPR), they provide additional experimental verification of the present computational results on *B. subtilis* MntR. These results are also in agreement with previously published thermal denaturation CD spectroscopy measurements, which show that Mn^2+^ ion binding induces thermal stabilisation of MntR [15]. These findings are supplemented by our own DSC studies of MntR thermal unfolding, which show that the Mn^2+^–MntR complex has both a higher melting temperature and a lower enthalpy of unfolding than the apo protein, and our CD spectroscopy measurements, which show an increase in the proportion of helical structures in MntR upon Mn^2+^ ion binding.

Several different structural parameters were considered as a quantitative measure of the observed difference between apo- and Mn^2+^-bound systems regarding the availability of the conformations adequate for DNA binding. The distance between the DNA-binding domains or DNA-binding helices, which is often used in the literature [13,14], is not significantly different for the Mn^2+^ and apo systems in our simulations (Figure S5 in SI). This is also true for the distance between the Cα atoms of the Lys41 residues in the two monomers [11,14]—there is no significant difference in this distance between the apo (37.8 ± 2.8 Å) and Mn^2+^ simulations (37.2 ± 1.9 Å) (Figure S8 in SI). The dihedral angle between the DNA-binding helices was found to be a much more relevant descriptor since it places emphasis on the relative orientation of DNA-binding helices (Figure 5). The proper orientation of these helices is likely crucial for DNA binding. Available structures of the MntR homologue IdeR in complex with DNA reveal that the DNA-binding helices bind in the major groove of the target DNA [18]. This is likely also true for the MntR protein from *B. subtilis*. For this type of binding to be possible, the DNA-binding helices need to be oriented towards each other in a very specific way. Therefore, the value of the dihedral angle between DNA-binding helices during simulations of the Mn^2+^ and apo systems offers a quantitative measure for the availability of the conformation adequate for DNA- binding. While values of the angle in the apo systems occasionally overlap with the values in the Mn^2+^ system, both the mean value and the standard deviation of the dihedral angle are significantly larger for the apo system.

Clustering analysis also pointed to the significance of studying dynamic properties for understanding the differences between Mn^2+^ and apo systems. The comparison of the representatives of the largest clusters reveals only a subtle difference in the positions of the DNA-binding helices (Figure 3). However, a look at the dynamics of the orientation of the helices paints a different picture, as the dihedral angle between the DNA-binding helices is consistently and significantly larger during the apo-simulations than during the simulations of Mn^2+^ systems (Figure 4).

As has been pointed out previously [10,11,13,14], the second Mn^2+^ binding site is likely crucial for fixing the DNA-binding domain in the proper orientation for DNA binding. We attempted to study the role of the first binding site by simulating the Zn^2+^–MntR complex. While the dihedral angle in the Zn^2+^–MntR simulations fluctuates even more than in the apo simulations, these results need further validation as the Zn^2+^ ions was parametrized using only non-bonded parameters.

In an attempt to imitate the binding of the second Mn^2+^ ion, we constructed mutants in which the coordinating residues of the second binding site were replaced with cysteines, with the idea that the resulting disulphide bond would imitate the binding of the second Mn^2+^ ion. 

Since the disulphide (S-S) bridge introduced by the D8C-E99C mutations should mimic Mn^2+^ binding, the D8C-E99C mutant should be locked in the conformation adequate for binding to DNA regardless of the presence or absence of Mn^2+^ ions. Indeed, MD simulations point to such a possibility, since the structural dynamics of the D8C-E99C mutant is more similar to the structural dynamics of the Mn^2+^ systems than to the apo system. However, besides changes in structural dynamics, i.e., conformational space, Mn^2+^ binding also induces polarisation of the electrostatic potential of the protein, in particular an increase in the otherwise already positive electrostatic potential of the DNA-binding helices (Figure 7). The same polarisation of electrostatic potential can not be observed in case of the D8C-E99C mutant, which could be the cause for its low DNA-binding affinity, despite the appropriate orientation of the DNA-binding helices causing locking of the conformational space by the S-S bridge.

The effect of the S-S bridge on structural dynamics was experimentally confirmed. Circular dichroism spectroscopy shows that the D8C-E99C mutant has a higher helical content than the wild-type protein in both its metal-free and metal-bound state (Figure 8, Table 2). An increase in helical content upon Mn^2+^ binding is not as pronounced for the mutant as for the wild-type protein, which is expected due to fact that Mn^2+^ binding to the second binding site is already mimicked by the S-S bridge. Circular dichroism spectroscopy did not allow us to determine whether the D8C-E99C mutant binds Mn^2+^ ions. DSC measurements, on the other hand, show that the enthalpy for unfolding of the mutant is smaller than for the wild-type protein. Even though the unfolding temperature is very similar (61.1° for the wild-type protein and 60.8° for the mutant), this implies that the mutant is less stable despite its higher helical content (Figure 9, Table 3). Although the binding of either the wild-type MntR or the D8C-E99C mutant to DNA in the presence or absence of Mn^2+^ ions could not be demonstrated with a native-PAGE assay, different electrophoretic mobility in native conditions was detected (Figure S4 in SI), consistent with molecular dynamics and CD spectroscopy results of altered electrostatic potential and secondary structure composition of the double mutant compared to the wild-type protein.

## 4. Materials and Methods

### 4.1. Preparation and Parametrization of Systems

Systems were prepared for simulation starting from five different crystal structures: two structures of the apo-protein (PDB IDs: 2HYF, 2HYG [14]), two structures of the Mn^2+^-bound protein (PDB IDs: 2F5F, 2F5C [11]) and a singly metalated form of MntR in the Zn^2+^-bound structure (PDB ID: 2EV6 [11]). Missing residues were added using the MODELLER software [19] within Chimera [20]. The N-terminal methionine was omitted, as previously published CD spectra suggest that it is likely excised [12]. Hydrogen atoms were added using the H++ web server [21,22,23]. The protonation was compared and some residues were manually re-protonated to assure symmetry between subunits of the homodimer, and to determine the effect of protonation on system dynamics. 

The structure of the D8C-E99C mutant was prepared starting from the crystal structure of the apo-protein (PDB ID: 2HYF [14]), and mutations were introduced using Chimera [19]. The structure was then protonated using the H++ web server [20,21,22]. No manganese ion is present in this structure, but the cysteine residues form a covalent disulfide bond.

The interaction between the protein and Mn^2+^ ions was parameterized using a bonded model, with bond and angle parameters derived from DFT calculations performed in Gaussian16 rev. B.01 [24]. The binuclear Mn^2+^ binding site was excised from the Mn^2+^ in protein complex (PDB ID: 2F5F [11]) in 13 different ways, generating systems of different sizes (Table S1 in SI). The systems were geometry optimized at the UB3LYP/aug-cc-pVDZ level of theory with Grimme’s [25] empirical correction for dispersion (GD3). All computations were performed in solvent (water) using the Polarizable Continuum Model (PCM). The optimized structures were submitted for the single point frequency calculation in Gaussian at the same level of theory. Parameters for bonds and angles were extracted from the frequency calculations using the VFFDT software [26]. Next, charges were calculated in Gaussian using the Merz–Singh–Kollman scheme [27]. Partial charges on the atoms were calculated using the RESP [27,28] procedure in Amber [29]. The parameters and charges for the Mn^2+^ binding sites can be found in Tables S3 and S4 of the SI.

The interaction of the Zn^2+^ ion with the protein in the simulation of the Zn^2+^–MntR complex was modelled using only non-bonded parameters, with the charge for the zinc (II) ion being +2, and van der Waals parameters from Li et al. [30].

The rest of the system was parameterized using the Amber ff14SB force field and the TIP3P water model. The program *tleap* which is part of the Amber20 software package was used to build the systems using the previously mentioned structures and parameter sets, apply periodic boundary conditions, and build a cubic water box around each protein so that the minimal distance between the protein and the edge of the box was 20 Å. Systems were neutralized with sodium ions.

### 4.2. Running MD Simulations

Systems were energy minimized in five cycles of 1000 steps each. The first 200 steps were performed using the steepest descent, and the next 800 steps using the conjugate gradient algorithm. In the first cycle the protein and Mn^2+^ ions were constrained with a harmonic potential of 100 kcal/(mol·Å^2^). In the second cycle, only the protein heavy atoms and Mn^2+^ ions were restrained using the same potential. In the third and fourth cycle only the protein backbone atoms were restrained—in the third with a harmonic potential of 100 kcal/(mol·Å^2^), and in the fourth with a harmonic potential of 50 kcal/(mol·Å^2^). In the fifth cycle the system was minimized without any constraints. After geometry optimization, systems were subjected to 500 ns of MD simulations. During the first 250 picoseconds (ps) of MD simulations, temperature was linearly increased from 0 K to 310 K, while, from 250 ps to 300 ps, temperature was kept constant using a Langevin thermostat with the heat coupling constant set to 1 ps. During the first 300 ps, volume was kept constant, and solute atoms were constrained using a harmonic potential with a force constant of 32 kcal/(mol·Å^2^). For the next 200 ps, the system was equilibrated at a constant temperature under constant pressure using the Langevin thermostat algorithm with the time constant for heat bath coupling set to 2 ps and isotropic position scaling. During the production phase, systems were simulated at constant pressure with a Langevin thermostat [31] and the time constant for heat bath coupling set to 1 ps. The time step of the simulation was 1 fs and structures were sampled every 100 ps. Periodic boundary conditions (PBC) were applied and electrostatic interactions were calculated using a particle mesh Ewald. Simulations were carried out using the Amber 20 simulation package.

### 4.3. Analysis of Simulations

Basic analyses such as RMSD, RMSF, radius of gyration, distance, angle and dihedral angle measurements as well as PCA analyses on the trajectories were performed in *cpptraj*, a program within the Amber20 program package [29]. Clustering was performed using the *gromos* algorithm and a cut-off of 1.8 Å within the Gromacs software package [32]. The cut-off was the same for all systems and it was selected according to trial and error so that neither a too large nor a too small number of clusters was obtained for any of the systems. In order to be able to compare the number of clusters for different systems, the same cut-off was used for all systems and all simulations. The obtained data were processed and plotted in R using the data.table [33] and ggplot2 [34] packages. Analyses of electrostatics were performed using the APBS software [35]. Trajectories were visualized using VMD [36] and Chimera [20]. 

For PCA and clustering analyses, the trajectories were stripped of the first three and last seven residues in each chain, as these residues fluctuate a lot during the simulations creating noise in the data. PCA was performed by extracting structures from 200 ns to 500 ns from trajectories of two apo systems prepared from the crystal structures 2HYF and 2HYG, and two Mn^2+^-bound systems prepared from the crystal structures 2F5F and 2F5C, merging them all, and then calculating covariance matrices on the Cartesian coordinates of the Cα protein backbone atoms. Factor loadings were calculated in R [37]. Clustering analysis was performed on each trajectory separately, starting at 20 ns.

The DichroCalc web server [38] was used to calculate CD spectra from molecular dynamics simulations. Spectra were calculated for the representatives of the five largest clusters from all simulated systems (Figure S9 in SI).

### 4.4. Protein Cloning, Expression and Purification

The plasmid *N-His_BmntR_pET-45b(+)* containing the gene for *Bacillus subtilis* MntR protein, with a sequence coding for 6 His and the recognition site for HRV-3C protease added at its N- terminal, was purchased from GenScript Biotech (Leiden, The Netherlands). The plasmid was subcloned using electroporation into *E. coli* cell strain BL21-CodonPlus(DE3)-RIL (Agilent Biotechnology, Santa Clara, CA, USA) which was used for expression of the protein. The expression was performed in LB medium containing 100 mg/mL of ampicillin. The cells were grown at 37 °C and 220 rpm to OD_600nm_~0.6 and expression was induced with 0.5 mM IPTG (Isopropyl β-D-1-thiogalactopyranoside) and proceeded overnight at 18 °C and 220 rpm. Cells were harvested at 5000× *g*, at 4 °C. 

Protein purification was performed at 4 °C. Cells were resuspended (in 10 mL per 1 g of cells) in 50 mM Na-phosphate buffer pH 7.5 containing 0.5 M NaCl, 10 mM imidazole and 10% of glycerol (buffer A), to which 0.1 mM PMSF was also added. High-pressure homogenizer (Avestin Emulsiflex C3, Avestin Inc., Ottawa, ON, Canada) was used to lyse the cells. Cell debris was separated from protein extract by centrifugation for 45 min at 15,000× *g* and 4 °C. Protein extract was loaded onto a column of 2.5 mL of Ni-NTA agarose (Protino, Macherey-Nagel, Düren, Germany) equilibrated in buffer A. Two column washing steps were performed—with 15 mL of buffer A and with 15 mL of buffer B (same as buffer A, but with 20 mM imidazole)—and bound proteins were eluted with 15 mL of buffer C (same as buffer A, but with 300 mM imidazole). His-tag was cleaved off with HRV-3C protease (Thermo Scientific, Waltham, MA, USA) during an overnight dialysis towards 1 L of 50 mM Hepes buffer pH 7.2, also containing 150 mM NaCl, 1 mM EDTA, 1 mM 2-mercaptoethanol and 10% of glycerol. One µmol of protease was added per 30 µmol of protein. For additional purification of the protein, size-exclusion chromatography (SEC buffer) was performed. Superdex 75 pg HiLoad 16/600 column (Cytiva Life Sciences, Marlborough, MA, USA) was equilibrated in 20 mM Hepes buffer pH 7.2 containing 200 mM NaCl and 10% of glycerol, and operated at 1 mL/min on the ÄKTA Pure FPLC system (Cytiva Life Sciences). Two milliliters of the concentrated sample were loaded onto the column, and fractions of 2 mL were collected. The final sample of purified protein (in SEC buffer) was stored in aliquots at −80 °C until use.

The purification was monitored using electrophoresis under denaturing conditions (SDS-PAGE) and protein concentration was determined utilizing protein’s molar extinction coefficient at 280 nm of 18,910 M^−1^ cm^−1^ (as calculated using the ProtParam tool at ExPasy.org).

Residues Asp8 and Glu99 were changed to cysteines with site-directed mutagenesis, one at a time, using Quik Change II Site-Directed Mutagenesis Kit (Agilent). The plasmid *N-His_BmntR_pET-45b(+)* was used as a template. Primers used are given in Table 4 (acquired from Macrogen Europe, Amsterdam, The Netherlands). The mutagenesis was checked by sequencing (Macrogen Europe, Amsterdam, The Netherlands). The double mutant of *B. subtilis* MntR was overexpressed and purified using the same procedure as the native protein.

### 4.5. Native Polyacrylamide Gel Electrophoresis Assay

For the native polyacrylamide gel electrophoresis (native-PAGE) assay, purified *B. subtilis* MntR protein and its double mutant (see section above) were mixed with the double-stranded oligonucleotide containing a recognition sequence in the mntH promotor (5′-AAAATAATTTGCCTTAAGGAAACTCTTT-3′, Macrogen Europe) in the presence and absence of manganese acetate. The concentration of protein in the mixture was 35 µM, of oligonucleotide 19 µM and of manganese acetate 74 µM. Four µL of each thus prepared mixture, together with protein alone in the same concentration, was loaded with no further treatment onto a 12.5% PhastGel and native-PAGE was performed on PhastSystem (Cytiva Life Sciences), with native buffer strips, according to the manufacturer’s instructions. The gel was stained with Coomassie Brilliant Blue R-250.

### 4.6. Ellman’s Reagent Reaction

To prove that the disulfide bridge is indeed formed in the D8C-E99C mutant of *Bacillus subtilis* MntR, Ellman’s reagent reaction was utilized. A protein sample (WT or mutant) was mixed with reaction buffer (0.1 M sodium phosphate buffer with 1 mM EDTA, pH 8.0) and Ellman’s reagent (5,5′-dithio-bis(2-nitrobenzoic acid), DTNB) and incubated for 15 min at room temperature (25 °C). The final concentration of protein in the incubation mixture was 4.5, 16 or 55 µM, and the final concentration of DTNB was 200 µM. After 15 min, absorbance of the incubation mixture at 412 nm was measured. A molar extinction coefficient of 14.150 M^−1^cm^−1^ [36] was used to calculate the concentration of 2-nitro-5-thiobenzoic acid (TNB, resulting from the reduction of Ellman’s reagent), which is equivalent to the concentration of free sulfhydryl groups in the mixture. In parallel, the same experiment was performed with protein samples treated with thiol-free reducing agent (tris(2-carboxyethyl)phosphine, TCEP), in 2× molar excess over protein, for 30 min at room temperature. Analysis of a control mixture with reaction buffer instead of protein was conducted in both cases (with and without the reducing agent), and its absorbance subtracted from the sample’s.

### 4.7. Circular Dichroism Measurements

Conformational analysis of *Bacillus subtilis* MntR and its Mn^2+^ complex was performed using circular dichroism (CD) spectroscopy. CD is a chiroptical method where the difference in absorption of left and right circularly polarized light is measured to calculate conformational motifs of proteins. CD spectra were recorded on a JASCO J-815 spectropolarimeter at room temperature, in SEC buffer, using 0.001 cm path quartz cuvettes with a scanning speed of 200 nm/min. The spectra were recorded as a mean value from three scans in the wavelength range 190–300 nm; the buffer spectrum was subtracted. The online service BeStSel [39,40] (Beta Structure Selection) was used to determine the secondary structure motifs of the studied proteins.

### 4.8. Differential Scanning Calorimetry Measurements

The thermodynamic profile of the apo-MntR and MntR–Mn^2+^ complex-unfolding process was measured with differential scanning calorimetry (DSC). The thermally induced conformational transition of the protein from the folded to unfolded state is usually observed with DSC. The excess heat capacity (Cp) of the protein solution is measured as a function of temperature (T). The protein unfolding is registered as an endothermic peak, with Tm being the center of the peak with a maximum Cp value. The peak width indicates cooperativity of the unfolding process, and the integration of the Cp vs. T gives the transition (unfolding) enthalpy ∆H°m. The ∆H°m calculated in this way represents the net value of endothermic contributions, such as the disruption of hydrogen bonds, aromatic stacking interactions, and exothermic ones, such as the disarranging of hydrophobic interactions [41]. The samples were prepared in SEC buffer. The DSC scans were performed on a TA instruments Nano DSC calorimeter with a 300 µL cell volume in the temperature range 25–110 °C at the scanning rate of 1 °C/min; buffer scans were subtracted prior to thermogram analysis. The recorded thermograms were analyzed with the NanoAnalyze Data Analysis (Version 3.11.0) software package. The thermal stability of the structure is assessed with Tm comparison. The Tm value presents a temperature point where half of the molecules are still in the folded state and half are unfolded. The cooperativity of the unfolding process is evaluated with the peak width at half its max height (∆T@1/2h) in °C.

## Figures and Tables

**Table 1:** Number of clusters found when performing clustering analysis using the gromos algorithm with a cut-off of 1.8 Å on trajectories of apo- and metal-bound MntR protein.

Simulation ^1^	Number of Clusters
Apo—PDB ID 2HYF	144
Apo—PDB ID 2HYG	172
Mn^2+^—PDB ID 2F5F	76
Mn^2+^—PDB ID 2F5C	113

^1^ PDB ID refers to the structure used as a starting point for preparing the MD simulation.

**Table 2:** The percentages of particular secondary structure motifs, as calculated using the Bestsel algorithm.

Motif/Sample	WT—apo	WT—Mn^2+^	D8C-E99C—apo	D8C-E99C—Mn^2+^
λ-helix	75.6%	93.3%	95.2%	100%
β-sheets	2.9%	0	4.8%	0
Turn	1.3%	6.7%	0	0
Disordered	20.3%	0	0	0

**Table 3:** Thermal unfolding characteristics of apo-MntR and its complex with Mn^2+^, as well as the D8C-E99C mutant and its complex with Mn^2+^, calculated from the DSC scans: Temperature (T_m_, °C), enthalpy (∆H, kJ·mol^−1^·K^−1^) and peak width at half its maximum height (∆T@1/2h, °C).

Sample	c (mg/mL)	Mw (kDa)	T_m_ (°C)	∆H_m_ (kJ/mol)	∆T@1/2h (°C)
WT—apo	1.115	16.7	61.09	104.80	4.96
WT—Mn^2+^	0.553	16.7	64.42	84.02	4.07
D8C-E99C—apo	0.94	16.7	60.77	54.01	3.95
D8C-E99C—Mn^2+^	0.94	16.7	61.19	51.55	3.95

**Table 4:** DNA sequences of specific primers used for amino-acid substitutions in *B. subtilis* MntR protein. Changes in sequence are denoted in bold.

Mutation	Primer	Sequence (5′→3′)
D8C	forward	CAGCATATAAATCTGTTCAATATAA**CA**TTCCATACTTGGTGTTGTCATGGGC
	reverse	GCCCATGACAACACCAAGTATGGAA**TG**TTATATTGAACAGATTTATATGCTG
E99C	forward	CCAGCTTAAATGATGTTCGATTCC**GCA**GACATCGTTATATATTTTCTCTTC
	reverse	GAAGAGAAAATATATAACGATGTC**TGC**GGAATCGAACATCATTTAAGCTGG

## Data Availability

Available on request.

## Supplementary Materials

The following supporting information can be downloaded at: https://www.mdpi.com/article/10.3390/ijms24020957/s1.
