# Supplementary material for: Structural Dynamics of the Bacillus subtilis MntR Transcription Factor Is Locked by Mn2+ Binding
Source: Int J Mol Sci. 2023 Jan 4;24(2):957. doi: 10.3390/ijms24020957 (PMC9861239; doi:10.3390/ijms24020957)
Supplement: Supplementary file 1 [file ijms-24-00957-s001.zip › ijms-2029078-supplementary.pdf]

International Journal of *Molecular Sciences*

MDPI

Supporting Information

Structural dynamics of the *Bacillus subtilis* MntR transcription factor is locked by Mn^{2+} binding

Zoe Jelić Matošević [1], Katarina Radman [1], Jolene Loubser [2], Ivo Crnolatac [3], Ivo Piantanida [3], Ignacy Cukrowski [2], Ivana Leščić Ašler [4] and Branimir Bertoša [1,*]

[1] Department of Chemistry, Faculty of Science, University of Zagreb, Horvatovac 102a, HR-10000 Zagreb, Croatia

[2] Department of Chemistry, Faculty of Natural and Agricultural Sciences, University of Pretoria, Lynnwood Road, Hatfield, Pretoria 0002, South Africa

[3] Division of Organic Chemistry & Biochemistry, Ruđer Bošković Institute, Bijenička cesta 54, HR-10000 Zagreb, Croatia

[4] Division of Physical Chemistry, Ruđer Bošković Institute, Bijenička cesta 54, HR-10000 Zagreb, Croatia

* Correspondence: bbertosa@chem.pmf.hr; Tel.: +385 1 4606 132

Table of contents:

1. Systems used for parametrization of MntR-Mn^{2+} interaction (Supplementary Table S1) S2
2. RMSD between crystal structures of MntR (Supplementary Table S2) S3
3. Parameters for the interaction of Mn^{2+} ions and MntR protein (Supplementary Table S3) S-3
4. Charges of residues coordinating Mn^{2+} ions in the MntR protein (Supplementary Table S4) S-4
5. RMSF of apo-MntR and the MntR-Mn^{2+} complex (Supplementary Figure S1) S-6
6. PC2 for all apo- and Mn^{2+}-simulations of MntR (Supplementary Figure 2) S-7
7. Distance between residues 8 and 99 (Supplementary figure S3) S-8
8. Native-PAGE electrophoresis (Supplementary figure S4) S-9
9. Distance between DNA-binding helices and domains (Supplementary Figure S5) S-10
10. Coordination of the Mn^{2+} atom in the MntR protein (Supplementary Figure S6) S-11
11. Coordination of the Zn^{2+} atom in the MntR protein (Supplementary Figure S7) S-12
12. Distance between the Cα atoms of Lys41 in MntR (Supplementary Figure S8) S-13
13. Computationally derived and CD spectra (Supplementary Figure S9) S-13
14. References S-14

Table S1. Systems of various sizes used for parametrization of the Mn^{2+}-protein interaction in the MntR protein. Structures were obtained by excising the Mn^{2+}-binding site of MntR from the Mn^{2+}-MntR complex (PDB ID: 2F5F [1]) in various ways.

ID	Nº of atoms	Structure	ID	Nº of atoms	Structure
**S1**	68	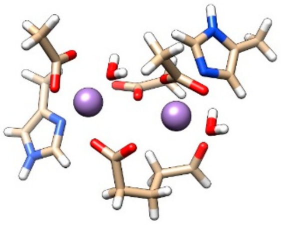	**S8**	76	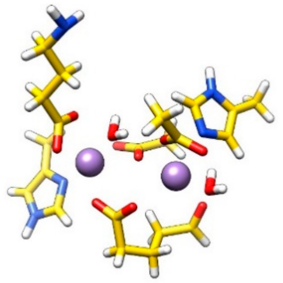
**S2**	81	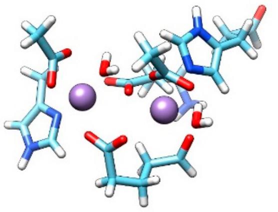	**S9**	76	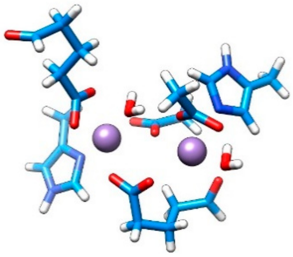
**S3**	83	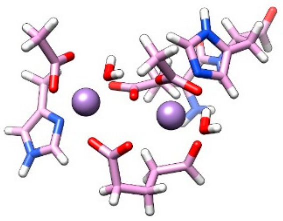	**S10**	78	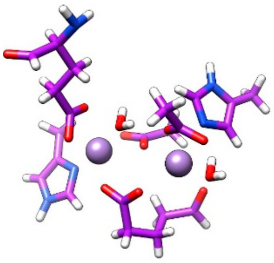
**S4**	70	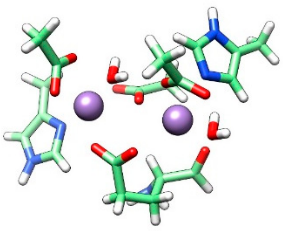	**S11**	73	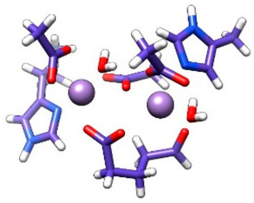
**S5**	73	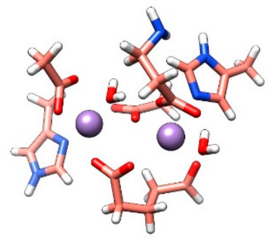	**S12**	73	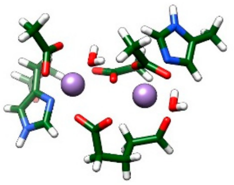

**S6**	73	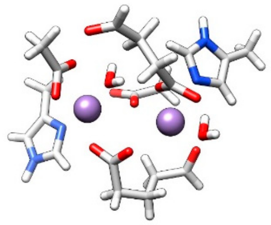	**S13**	75	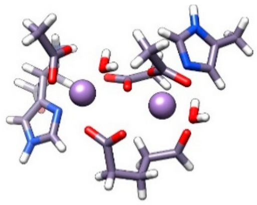
**S7**	75	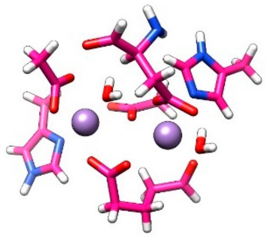			

Table S2. The root mean square deviation between crystal structures of the *Bacillus subtilis* MntR protein in its Mn^{2+}-bound (PDB IDs 2F5F, 2F5C [1]) and its apo-form (PDB IDs 2HYF, 2HYG [2]) calculated for the backbone of the whole dimer, but including only residues 4 to 135, as these are present in all crystal structures. For the structure 2HYF biological assembly 2 was used, while for 2HYG biological assembly 1 was used.

	**2F5F**	**2F5C**	**2HYF – BA2**	**2HYG – BA1**
**2F5C**	0.89			
**2HYF – BA2**	2.14	1.72		
**2HYG – BA1**	2.66	2.02	1.77	
**2EV6**	0.84	1.39	2.35	3.12

Table S3. The parameters for the Mn^{2+} binding site of *B. subtilis* MntR.

**BOND**			**ANGLE**			**ANGLE**		
atoms			atoms			atoms		
M2-Z3	18.51	22.874	CC-Y3-M1	38.707	129.15	Y8-M2-Y9	19.066	109.74
Y1-M1	21.28	22.455	CO-Y1-M1	41.368	91.72	Y8-M2-Z2	25.188	93.70
Y2-M1	19.38	22.921	CO-Y2-M1	64.916	89.94	Y8-M2-Z3	42.863	87.86
Y3-M1	49.35	21.794	CO-Y4-M1	19.465	125.81	Y9-M2-Y6	19.800	85.98
Y4-M1	31.25	21.540	CO-Y5-M1	69.691	89.99	Y9-M2-Z2	24.311	156.14
Y5-M1	7.26	23.707	CO-Y6-M1	89.929	94.48	Y9-M2-Z3	20.166	88.30
Y6-M1	27.71	22.498	CO-Y6-M2	31.772	143.96	Z2-M2-Z3	47.186	88.17
Y6-M2	27.57	21.586	CO-Y8-M2	27.655	131.19	2C-CO-Y1	70.0	117.00
Y8-M2	36.74	21.254	CO-Y9-M2	22.247	131.60	2C-CO-Y2	70.0	117.00
Y9-M2	47.29	20.888	CR-Z2-M2	78.640	122.47	2C-CO-Y4	70.0	117.00
Z2-M2	18.34	21.805	M1-Y3-CR	36.91	123.81	2C-CO-Y5	70.0	117.00
C -Z1	570.0	1.229	M2-Y6-M1	27.856	121.54	2C-CO-Y6	70.0	117.00
CC-Y3	410.0	1.394	M2-Z2-CV	92.463	130.85	2C-CO-Y8	70.0	117.00
CO-Y1	656.0	12.500	M2-Z3-HL	24.247	104.15	2C-CO-Y9	70.0	117.00
CO-Y2	656.0	12.500	Y1-M1-Y2	73.783	58.18	CC-CV-Z2	70.0	120.00
CO-Y4	656.0	12.500	Y1-M1-Y3	22.947	104.91	CC-Y3-CR	70.0	117.00

CO-Y5	656.0	12.500	Y1-M1-Y4	24.406	151.35	CR-Z2-CV	70.0	117.00
CO-Y6	656.0	12.500	Y1-M1-Y5	44.215	86.99	CT-CC-Y3	70.0	120.00
CO-Y8	656.0	12.500	Y1-M1-Y6	35.033	94.25	CW-CC-Y3	70.0	120.00
CO-Y9	656.0	12.500	Y2-M1-Y3	23.739	100.99	NA-CR-Z2	70.0	120.00
CR-Z2	488.0	1.335	Y2-M1-Y4	33.126	95.39	O2-CO-Y8	80.0	126.00
Y3-CR	488.0	1.335	Y2-M1-Y5	35.212	144.79	CX-C -Z1	80.0	120.40
Z2-CV	410.0	1.394	Y2-M1-Y6	56.005	116.20	Y2-CO-Y1	80.0	126.00
Z3-HL	553.0	0.9572	Y3-M1-Y4	30.012	89.92	Y3-CR-H5	50.0	120.00
Y7-HL	553.0	0.9572	Y3-M1-Y5	33.989	92.32	Y3-CR-NA	70.0	120.00
			Y3-M1-Y6	27.314	142.79	Y4-CO-Y9	80.0	126.00
			Y4-M1-Y5	30.964	117.27	Y6-CO-Y5	80.0	126.00
			Y4-M1-Y6	49.631	87.81	Z1-C -N	80.0	122.90
			Y5-M1-Y6	66.372	56.62	Z2-CR-H5	50.0	120.00
			Y6-M2-Z2	43.018	98.47	Z2-CV-H4	50.0	120.00
			Y6-M2-Z3	30.265	173.30	HL-Z3-HL	100.	104.52
			Y8-M2-Y6	21.747	90.78	HL-Y7-HL	100.	104.52

Table S4. The atom names and charges for the Mn^{2+} binding site of *B. subtilis* MntR.

**GLU**	**GU2**			**GLU**	**GU1**	
**atom name** *(PDB)*	*(force field)*	**charge**		**atom name** *(PDB)*	*(force field)*	**charge**
N	N	-0.516300		N	N	-0.516300
H	H	0.293600		H	H	0.293600
CA	CX	0.039700		CA	CX	0.039700
HA	H1	0.110500		HA	H1	0.110500
CB	2C	-0.053830		CB	2C	0.140007
HB2	HC	0.039483		HB2	HC	-0.019300
HB3	HC	0.039483		HB3	HC	-0.019300
CG	2C	-0.102578		CG	2C	0.097607
HG2	HC	0.072351		HG2	HC	-0.044500
HG3	HC	0.072351		HG3	HC	-0.044500
CD	CO	0.535244		CD	CO	0.685205
OE1	Y9	-0.566656		OE1	Y1	-0.559189
OE2	Y4	-0.545551		OE2	Y2	-0.694706
C	C	0.536600		C	C	0.536600
O	Z1	-0.538560		O	O	-0.581900

**GLU**	**GU3**			**ASP**	**AP1**	
**atom name** *(PDB)*	*(force field)*	**charge**		**atom name** *(PDB)*	*(force field)*	**charge**
N	N	-0.516300		N	N	-0.516300

	H	H	0.293600		H	H	0.293600
	CA	CX	0.039700		CA	CX	0.038100
	HA	H1	0.110500		HA	H1	0.088000
	CB	2C	0.140007		CB	2C	-0.030300
	HB2	HC	-0.019300		HB2	HC	-0.012200
	HB3	HC	-0.019300		HB3	HC	-0.012200
	CG	2C	0.097607		CG	CO	0.663163
	HG2	HC	-0.044500		OD1	Y8	-0.630164
	HG3	HC	-0.044500		OD2	O2	-0.688784
	CD	CO	0.467374		C	C	0.536600
	OE1	Y5	-0.589654		O	O	-0.581900
	OE2	Y6	-0.401108				
	C	C	0.536600				
	O	O	-0.581900				

	**HIS**	**HD1**			**HIS**	**HE1**	
**atom name**	*(PDB)*	*(force field)*	**charge**	**atom name**	*(PDB)*	*(force field)*	**charge**
	N	N	-0.415700		N	N	-0.415700
	H	H	0.271900		H	H	0.271900
	CA	CX	0.018800		CA	CX	-0.058100
	HA	H1	0.088100		HA	H1	0.136000
	CB	CT	-0.009200		CB	CT	-0.006638
	HB2	HC	0.040962		HB2	HC	0.036700
	HB3	HC	0.040962		HB3	HC	0.036700
	CG	CC	0.057384		CG	CC	0.260800
	ND1	NA	-0.057102		ND1	Y3	-0.220610
	HD1	H	0.310384		CE1	CR	0.000000
	CE1	CR	-0.258548		HE1	H5	0.141181
	HE1	H5	0.254468		NE2	NA	-0.149019
	NE2	Z2	-0.068230		HE2	H	0.341589
	CD2	CV	-0.182759		CD2	CW	-0.283798
	HD2	H4	0.166071		HD2	H4	0.208386
	C	C	0.597300	C	C		0.597300
	O	O	-0.567900	O	O		-0.567900

	**MN**	**MN2**			**WAT**	**WT1**	
**atom name**	*(PDB)*	*(force field)*	**charge**	**atom name**	*(PDB)*	*(force field)*	**charge**
	MN	M2	1.063.580		O	Z3	-0.818191
					H1	HL	0.396213

	**MN**	**MN1**	
**atom name**	*(PDB)*	*(force field)*	**charge**
	MN	M1	0.890700

H2 HL 0.396213

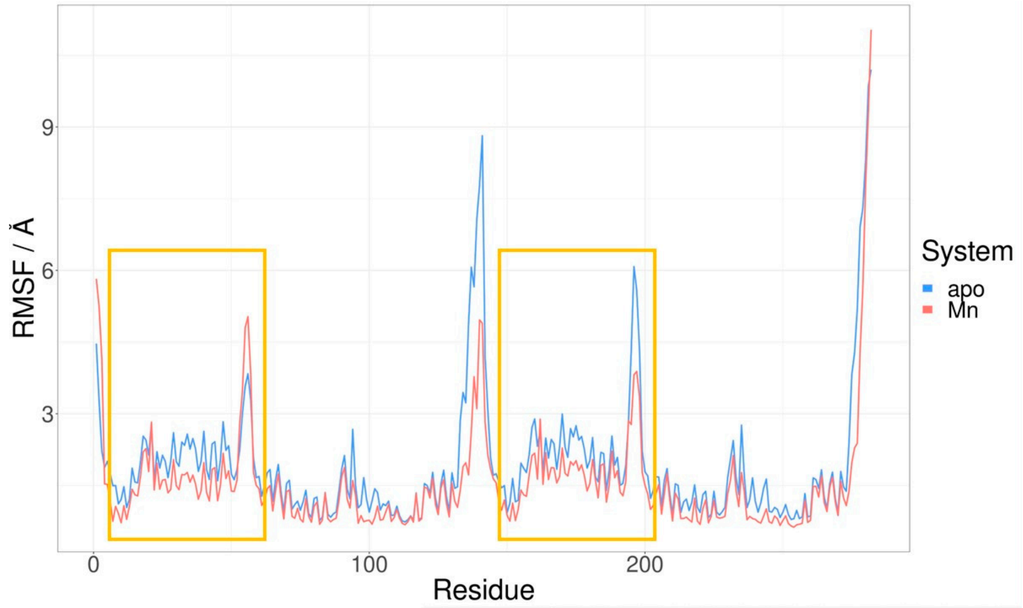

Figure S1. Root mean square fluctuations calculated for each residue in the MntR protein in the apo- and Mn^{2+}-simulation. The DNA-binding domains are highlighted in yellow.

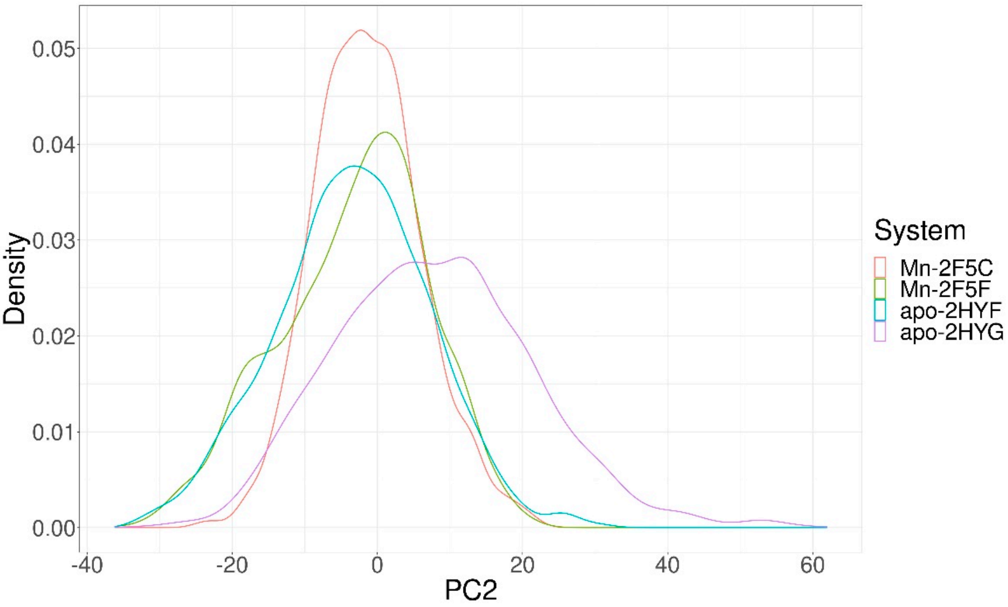
Density
PC2
System
Mn-2F5C
Mn-2F5F
apo-2HYF
apo-2HYG
0.00
0.01
0.02
0.03
0.04
0.05
-40
-20
0
20
40
60

(**a**)

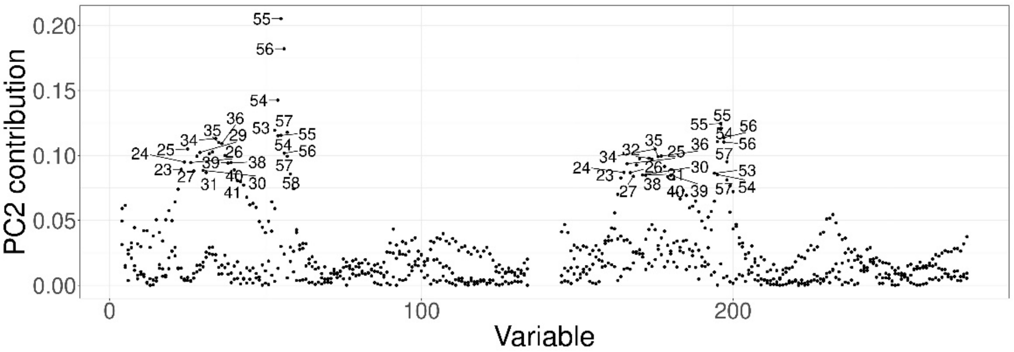
PC2 contribution
Variable
0.00
0.05
0.10
0.15
0.20
0
100
200

(**b**)

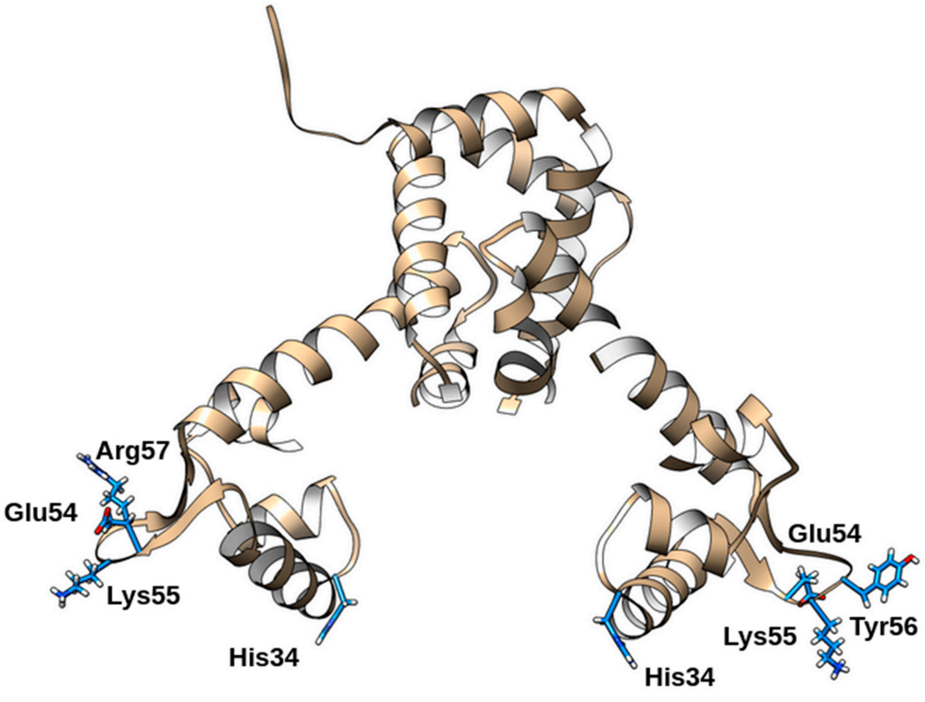
Arg57
Glu54
Lys55
His34
Glu54
Tyr56
Lys55
His34

(**c**)

Figure S2. (**a**) The projection of four trajectories of the MntR protein in its apo- (red and green) and Mn^{2+}-bound forms (blue and purple) onto principal component 2 obtained by principal component analysis of the cartesian coordinates of the Cα backbone protein atoms. (**b**) Factor loading for principal component 2 - the labelled points are counted for each chain of the dimer individually. (**c**) Residues represented in the factor loading are shown in sticks. Most of them are placed in the wing motifs of the DNA-binding domain. The projections of the trajectories onto the second principal component (PC2) are overlapping and all trajectories take up a similar range in the PC2 space. The variance in PC2, thus, likely represents the most pronounced global movements of the protein regardless of the presence of Mn^{2+} ions. Factor loadings for PC2 reveal that the largest contributions to these global movements come from the beta-sheet wing motifs in the DNA-binding domain.

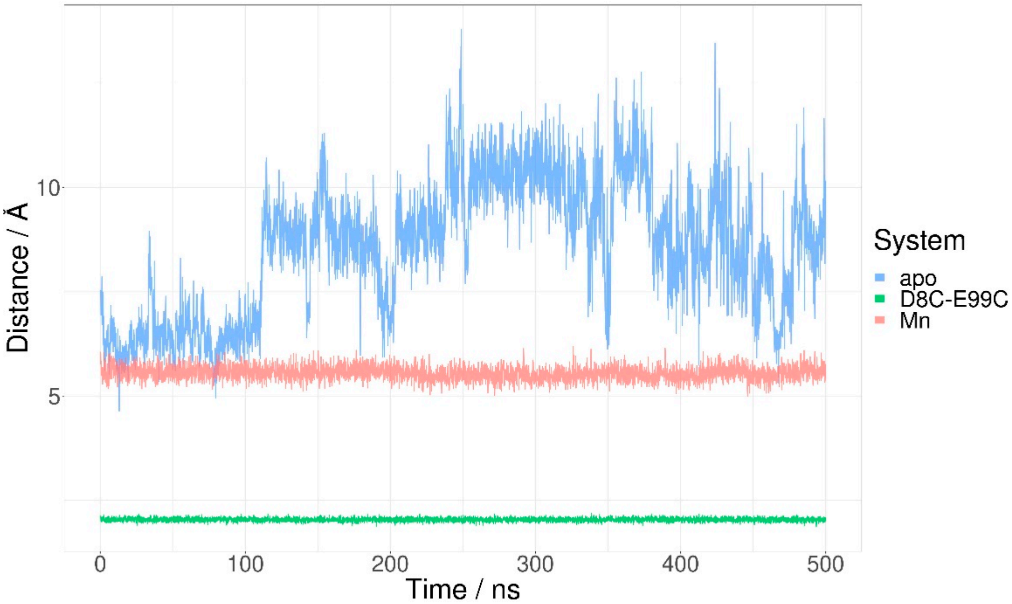

Figure S3. The length of the disulphide bond between residues 8 and 99 of the D8C-E99C mutant and the distance between the corresponding atoms of Asp8 (the CG atom) and Glu99 (The CG atom) in simulations of the apo- and Mn^{2+}-systems.

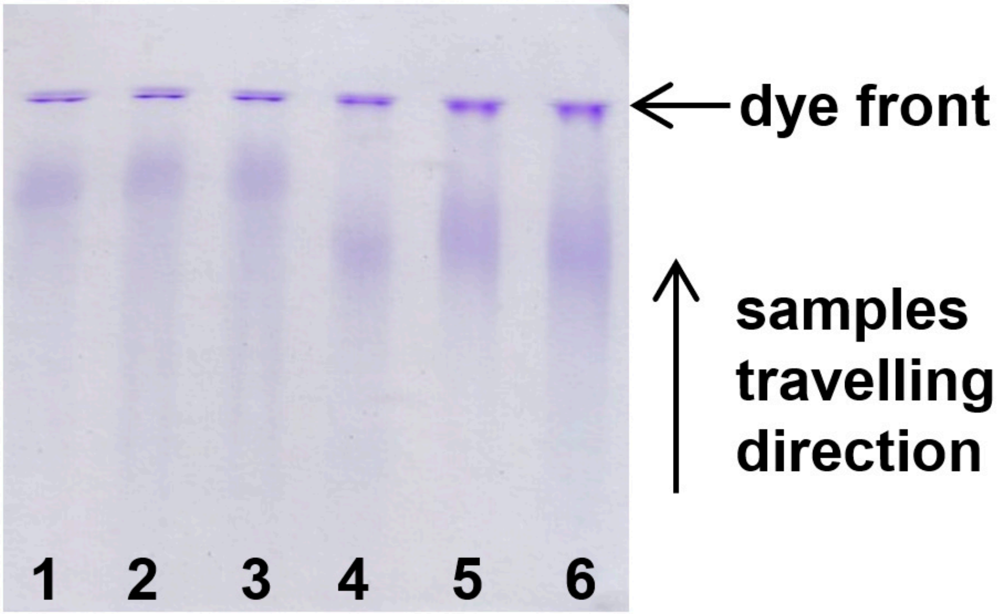

Figure S4. The native-PAGE assay of *B. subtilis* MntR protein, wild-type and D8C-E99C mutant, on a 12.5 % gel. Lanes 1, 2 and 3: wild-type MntR alone, with DNA and with both Mn^{2+} and DNA, respectively. Lanes 4, 5 and 6: mutant MntR alone, with DNA and with both Mn^{2+} and DNA, respectively.

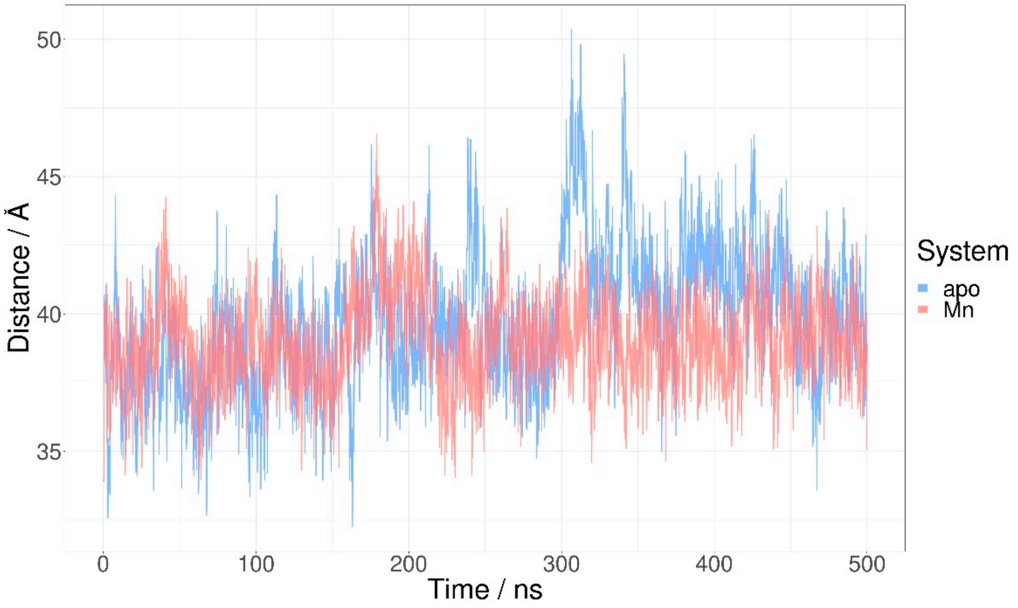

(**a**)

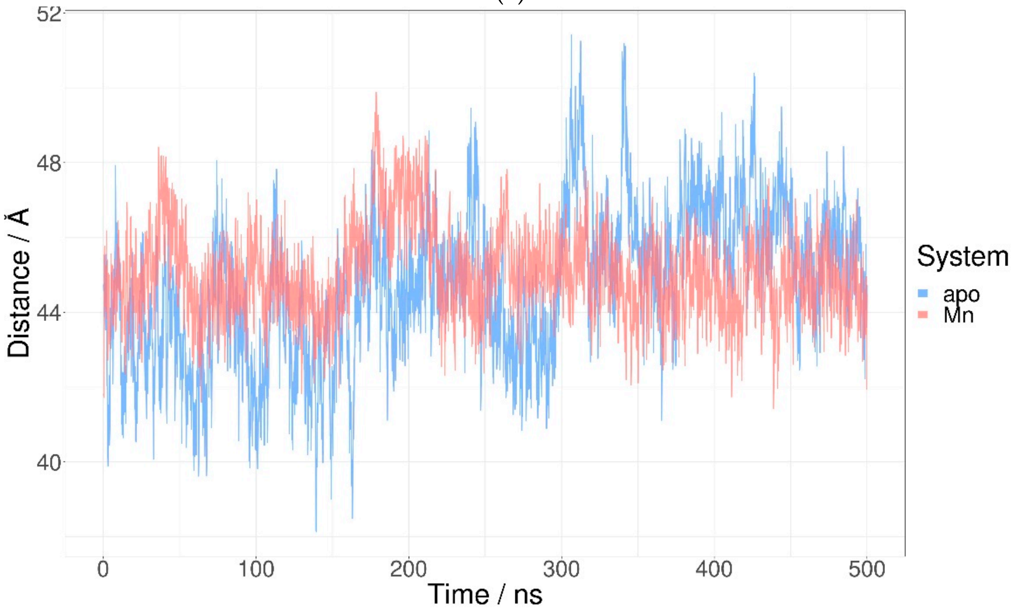

(**b**)

Figure S5. (**a**) The distance between the DNA-binding helices of the MntR protein. (**b**) The distance between the DNA-binding domains of the MntR protein.

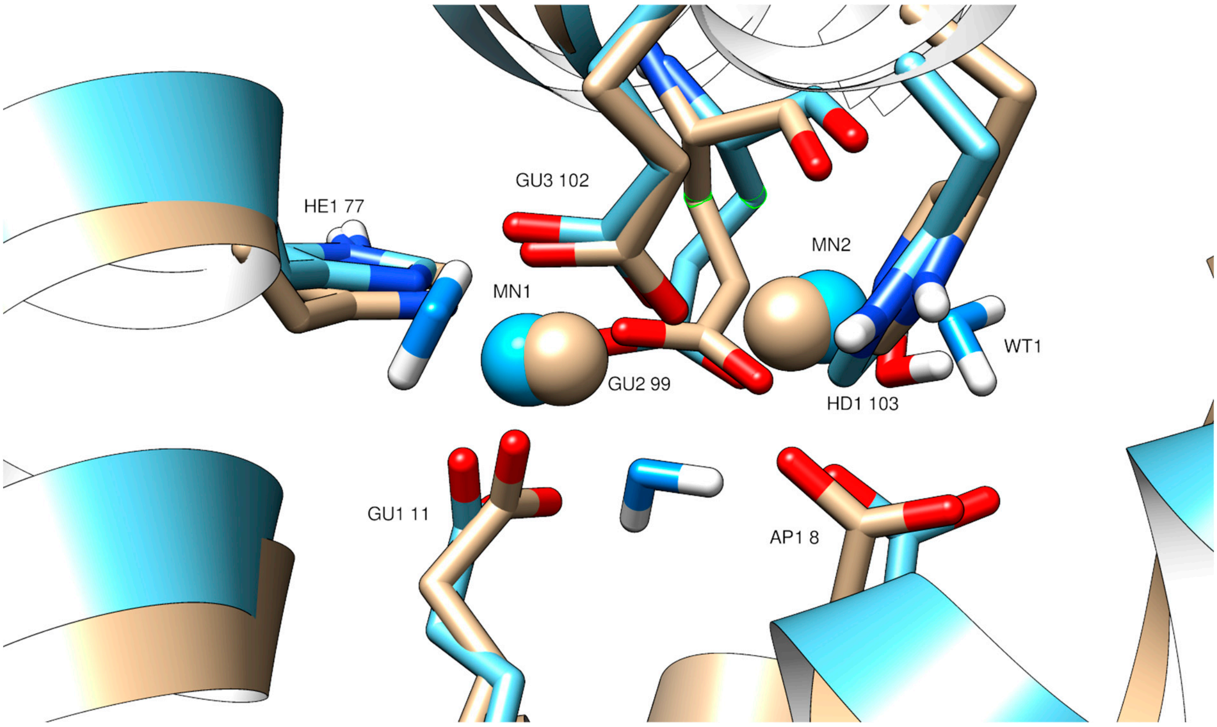

Figure S6. The Mn^{2+} binding site of *B. subtilis* MntR in the crystal structure (PDB ID: 2F5F [1], blue) and after 500 ns of molecular dynamics simulation (tan).

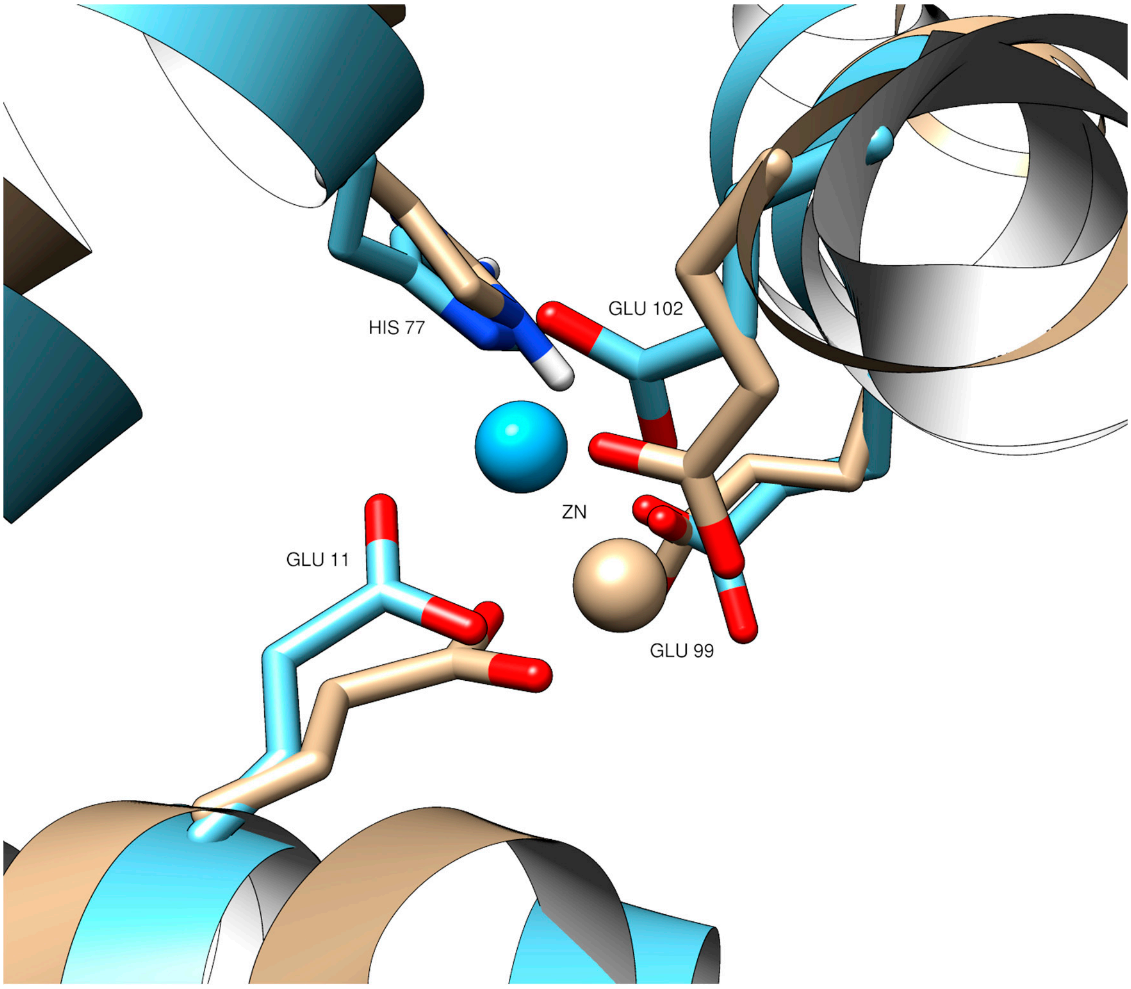

Figure S7. The Zn^{2+} binding coordination of *B. subtilis* MntR in the crystal structure (PDB ID: 2EV6 [1], blue) and after 500 ns of molecular dynamics simulation (tan).

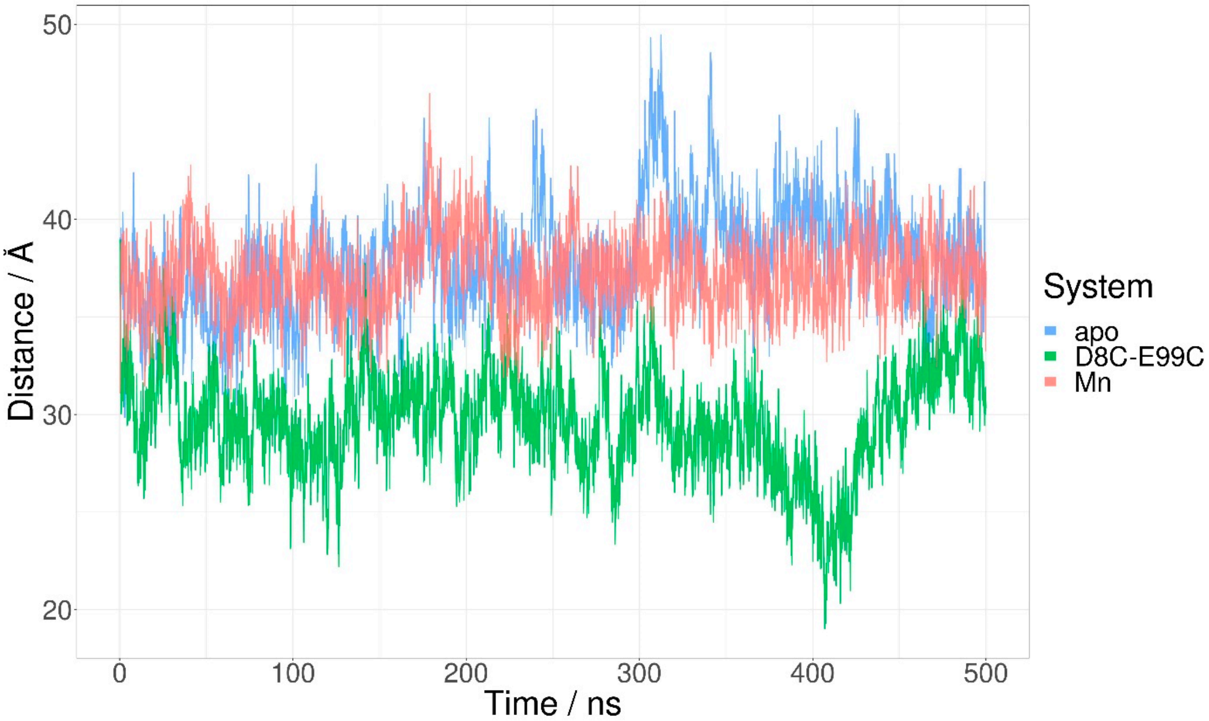

Figure S8. The distance between the Cα atoms of Lys41 in the two subunits of the MntR dimer for the apo- (blue), Mn^{2+}- (pink) and D8C-E99C-mutant (green) systems.

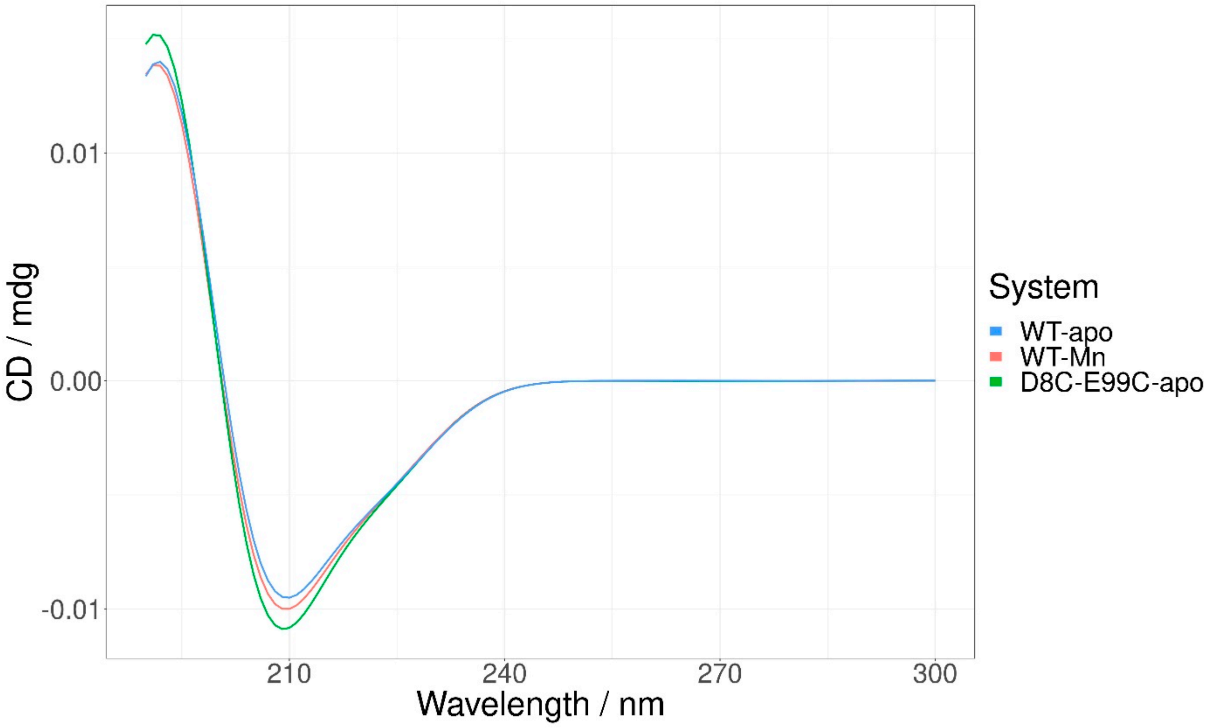

(**a**)

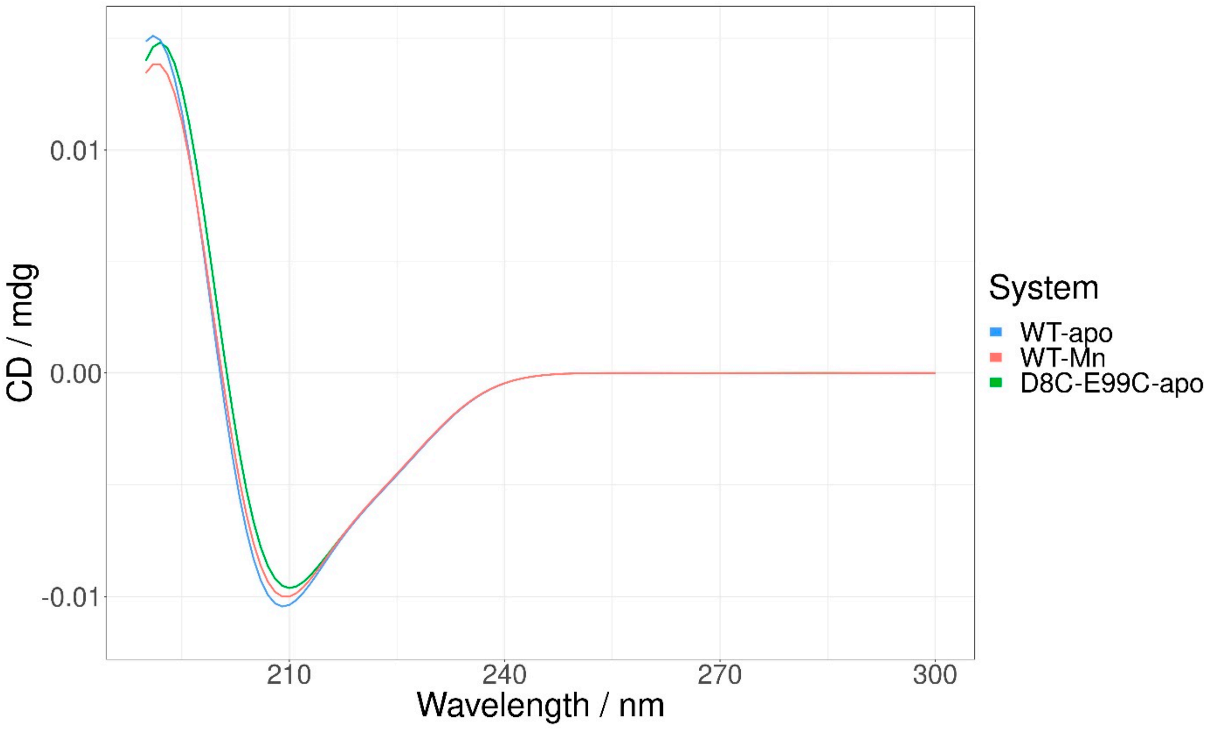

(**b**)

Figure S9. The computationally derived CD spectra of the apo- (blue), Mn^{2+}- (pink) and D8C-E99C-mutant (green) structures calculated using the DichroCalc server [3]. Spectra are calculated either on (**a**) the structure which is the representative of the largest cluster for each simulation or (**b**) on the structure which is the representative of the largest (WT-Mn), the third largest (WT-apo) and the second largest cluster (D8C-E99C-apo).

References

1. Kliegman, J.I.; Griner, S.L.; Helmann, J.D.; Brennan, R.G.; Glasfeld, A. Structural Basis for the Metal-Selective Activation of the Manganese Transport Regulator of *Bacillus Subtilis*. *Biochemistry* **2006**, *45*, 3493–3505, doi:10.1021/bi0524215.
2. DeWitt, M.A.; Kliegman, J.I.; Helmann, J.D.; Brennan, R.G.; Farrens, D.L.; Glasfeld, A. The Conformations of the Manganese Transport Regulator of Bacillus Subtilis in Its Metal-Free State. *Journal of Molecular Biology* **2007**, *365*, 1257–1265, doi:10.1016/j.jmb.2006.10.080.
3. Jasim, S.B.; Li, Z.; Guest, E.E.; Hirst, J.D. DichroCalc: Improvements in Computing Protein Circular Dichroism Spectroscopy in the Near-Ultraviolet. *Journal of Molecular Biology* **2018**, *430*, 2196–2202, doi:10.1016/j.jmb.2017.12.009.
